# Vision before and after scharioth macular lens implantation in patients with AMD: an electrophysiological study

**DOI:** 10.1007/s10633-020-09814-8

**Published:** 2021-01-03

**Authors:** Jan Kremláček, Jana Nekolová, Markéta Středová, Jana Langrová, Jana Szanyi, Miroslav Kuba, Zuzana Kubová, František Vít, Petr Voda, Martina Veselá, Naďa Jirásková

**Affiliations:** 1 Department of Medical Biophysics, Faculty of Medicine in Hradec Kralove, Charles University, Simkova 870, 500 38 Prague, Hradec Kralove, Czech Republic; 2 Department of Pathological Physiology, Faculty of Medicine in Hradec Kralove, Charles University, Prague, Czech Republic; 3 Department of Ophthalmology, Faculty of Medicine in Hradec Kralove, University Hospital Hradec Kralove, Charles University, Prague, Czech Republic

**Keywords:** Scharioth macular lens, Maculopathy, Age-related macular degeneration, Oddball ERPs, Motion-onset VEPs, Pattern-reversal VEPs, P3b

## Abstract

**Background:**

For patients with age-related macular degeneration (AMD), a special intraocular lens implantation partially compensates for the loss in the central part of the visual field. For six months, we evaluated changes in neurophysiological parameters in patients implanted with a “Scharioth macula lens” (SML; a center near high add + 10 D and peripheral plano carrier bifocal lens designed to be located between the iris and an artificial lens).

**Methods:**

Fourteen patients (5 M, 9 F, 63–87 years) with dry AMD were examined prior to and at 3 days after, as well as 1, 2, and 6 months after, implantation using pattern-reversal, motion-onset, and cognitive evoked potentials, psychophysical tests evaluating distant and near visual acuity, and contrast sensitivity.

**Results:**

Near visual acuity without an external aid was significantly better six months after implantation than before implantation (Jaeger table median (lower; upper quartile): 4 (1; 6) vs. 15 (13; 17)). Distant visual acuity was significantly altered between the pre- (0.7 (0.5; 0.8) logMAR) and last postimplantation visits (0.8 (0.7; 0.8) logMAR), which matched prolongation of the P100 peak time (147 (135; 151) ms vs. 161 (141; 166) ms) of 15 arc min pattern-reversal VEPs and N2 peak time (191.5 (186.5; 214.5) ms vs. 205 (187; 218) ms) of peripheral motion-onset VEPs.

**Conclusion:**

SML implantation significantly improved near vision. We also observed a slight but significant decrease in distant and peripheral vision. The most efficient electrophysiological approach to test patients with SML was the peripheral motion-onset stimulation, which evoked repeatable and readable VEPs.

**Supplementary Information:**

The online version containssupplementary material available at (10.1007/s10633-020-09814-8).

## Introduction

A loss of central vision impairs a person’s ability to carry out everyday activities and causes increased anxiety, depression, and trauma in the elderly population. It also decreased quality of life comparably to other systemic diseases such as cancer, ischemic heart disease, and stroke [1] and increases the risk of death more than twofold [2]. The most common form of focal macular retinopathy or maculopathy is age-related macular degeneration (AMD). AMD affects millions of individuals among the aging population worldwide and is the leading cause of legal blindness in all high-income EU countries [3]. Approximately 90% of patients suffering from AMD have a “dry” form of AMD with no short-term prospects of drug-related relief.

To help patients with AMD, various optical or optoelectronic external low-vision aids can be used to enhance images so that they are recognizable by the perimacular part of the retina. Frequently used optical devices enlarge the near visual scene. Such external devices have several limitations for visual perception; the most important limitations include (1) the reduced role of eye movement in visual scene exploration, (2) disturbances of the vestibular ocular reflex, and (3) impaired depth perception (4) necessity to carry and take care of the aid [4].

Currently, AMD patients have the option of using intraocular telescopes implanted in the anterior segment of the eye, which can surpass disadvantages (1), (2), and (4). While these telescopes still limit the visual field of view by 20–24°, they can substantially improve quality of life [4–7]. This limitation led to progression from a Galilean type toward a mirror lens telescope (Cassegrain type) and opened peripheral vision [8]; however, it reduced contrast and luminance [9]. The disadvantage of the implanted aids is that they cannot be adjusted for a desired focus/magnification.

Recent developments have shown that an add-on bifocal aspheric lens with unmagnified periphery and an addition of + 10 diopters in the central portion measuring 1.5 mm in dia (“Scharioth macula lens”–SML, Medicontur International, Geneva, Switzerland) [10] could improve near vision and maintain distant vision without visual field reduction [11–13]. The main benefit of such a high add is that a closer working distance may be used (relative distance magnification) so that patients are able to read smaller sized print with less reliance on external magnifiers such as hand or stand magnifiers.

A schematic illustration of the function of the SML is shown in Fig. 1.

The aim of this work is to describe the effect of SML implantation on brain activity using standard electrophysiological examination in patients with AMD. For this aim, we purposely selected ISCEV standard pattern-reversal stimuli [14] and extended them with motion-onset stimuli of low-contrast and low spatial frequency radial structure to cover a wider range of visual processing [15, 16] and with oddball event-related potentials to explore the cognitive part of visual processing [17].

The addition of a simple + 10 D spherical correction would significantly reduce visual acuity at the observation distance of 60 cm in an emmetropic subject. Consequently, an amplitude decrease and a prolongation of the P100 peak time can be expected, especially for a structure with a higher spatial frequency [18]. However, the SML is bifocal, and the manufacturer assumes that under optimal conditions, the light will travel through the implant rim without magnification (see Fig. 1b). In such a situation, the SML implantation should not affect the peak P100.

Due to the unstable visual fixation in patients with AMD, we included motion-onset stimulation in the examination protocol. This stimulation should be more robust to fluctuations in patients’ fixation due to low spatial frequency and concentric radial motion. At the same time, motion stimulation effectively and objectively examines the peripheral vision [19]. We expected that the motion-onset stimulation responses would be more robust, and the SML implantation should not alter the N160 peak parameters.

Postoperative visual rehabilitation is part of a practice to improve vision with the SML. For one month, the patients are intensively engaged in visual tasks. Using cognitive potentials, we tested whether patients' performance in the visual domain will increase.

Suppose the SML aid and the subsequent rehabilitation are effective. In that case, there should be no decrease in response to the pattern-reversal or motion-onset stimulation. Still, we may expect an improvement in cognitive response.

## Methods

### Patients

The selection criteria for implantation were based on recommendations of the SML manufacturer (Medicontur International, Geneva, Switzerland): age over 55 years, best-corrected distant visual acuity (BCVA) from 0.4 to 1.3 logMAR (i.e., 0.4–0.05 in decimal fraction), stabilized maculopathy, and a pseudophakic eye. Near visual acuity (NVA) was tested preoperatively with + 6.0 diopters from 15 cm, which is known to be equal to uncorrected NVA postoperatively and should be Jaeger table 6 or better. The exclusion criteria were a photopic pupil size less than 2.5 mm, severe zonulopathy, an anterior chamber depth less than 2.8 mm, narrow-angle or severe ocular pathology, or previous retinal surgery.

Patient selection, surgery, clinical care, and some of the examinations were conducted at the Department of Ophthalmology, University Hospital in Hradec Kralove, Czech Republic. At Department of Pathological Physiology, Charles University-Faculty of Medicine in Hradec Kralove, Czech Republic, patients underwent the electrophysiological examinations. The study was approved by the Ethics Committee of University Hospital in Hradec Kralove. All tenets of the Declaration of Helsinki were followed, and all patients provided informed consent.

The SML was implanted in the better seeing eye by one of the authors (surgeon N.J.). After pupil dilatation and application of topical anesthesia, a 2.2 mm incision was made. The anterior chamber was filled with an ophthalmic viscosurgical device, and the SML was implanted using a cartridge. After implantation, proper positioning of the haptics in the ciliary sulcus and IOL centration were checked. Finally, the ophthalmic viscosurgical device was removed, and the incision was hydrated.

All the patients underwent standard postoperative treatment with a topical antibiotic for 1 week and a topical steroid for 1 month. We informed patients that sharp vision is achieved at a very near distance of 10–15 cm and advised them to read text from the largest to the smallest type without glasses or external magnifiers at least twice a day for 10 min per session. To facilitate postoperative vision, all the patients performed a half an hour of a visual rehabilitation under supervision during the first 3 weeks.

All patients were examined at approximately 27 days (median from a range 5–49 days) before SML implantation and then at the third day and the first, second, and sixth months after SML implantation.

### Procedure

On every day of the examination, the BCVA was measured from 6 m using the Early Treatment Diabetic Retinopathy Study (ETDRS) chart [20], and the uncorrected NVA was measured by Jaeger tables–a printed text of increasing median letter size from 0.37 (J1) mm to 50.1 mm (J24) [21]. The table was held by the patient, and the smallest print that the patient could read determined his/her NVA. Reading from a distance of 175 mm, the text of size J15, J10, J5, and J1 corresponds to logMAR 1.3, 0.9, 0.6, 0.4,^1^ respectively.

Before implantation, the NVA was tested with added + 6 D (NVA + 6 D). These readings were performed at University Hospital.

At the Faculty of Medicine, electrophysiological examinations were performed in a darkened, sound-attenuated, electromagnetically shielded room with a background luminance of 0.1 cd/m^2^. During the experiment, patients were sitting in a comfortable dental chair with neck support to reduce muscle artifacts. The correct fixation was monitored via a near-infrared camera.

Before electrophysiological acquisition, Michelson luminance contrast sensitivity was determined using Landolt C with an outer diameter 480′ on a computer monitor with the Freiburg Visual Acuity Test (4 choices, 24 trials, and screen resolution of 1024 × 768 pixels) [22].

All stimuli for the electrophysiological tests and the contrast sensitivity test were presented on a 21″ computer monitor (Vision Master Pro 510, Iiyama, Japan) subtending 37° × 28° of the visual field from an observation distance of 0.6 m. The visual stimuli for VEPs were presented using the Visual Stimulus Generator 2/5 (CRS ltd., UK) at a vertical refresh frequency of 105 Hz. The ERP stimuli were presented using Psychtoolbox 3 [23] at a vertical refresh frequency of 75 Hz. The recorded epochs were synchronized with a backward trace of the monitor’s electron beam just before the first video frame of an appropriate stimulus change. The recordings were performed monocularly, and the implanted eye results were analyzed and reported.

VEPs/ERPs were recorded from 6 unipolar derivations (O_Z_, P_Z_, C_Z_, F_Z_, and O_L_, O_R_-5 cm left and right of the O_Z_) with a right earlobe reference (A_2_). The minimum set of recording derivations was chosen based on a previous topographical study concerning the scalp distribution of motion-onset VEPs [24]. The ground electrode was connected to the reference. All electrode impedances were kept below 10 kΩ. The signal was amplified in the frequency band of 0.3–100 Hz (PSYLAB, System 5, Contact Precision Instruments, USA).

#### Pattern-reversal VEPs

Over 20 s, forty reversals of a high-contrast black and white checkerboard pattern were used to evoke pattern-reversal VEPs. Two variants of the checkerboard stimulation were used: with check sizes of 60′ (*PR-VEP* *60′*) and 15′ (*PR-VEP 15′*). Each VEP variant was examined twice. The mean luminance of 17 cd/m^2^ was constant during the examination. Patients’ task was to keep their gaze on the fixation cross during the recording.

EEG poststimulus epochs of 440 ms duration were sampled at 500 Hz. Epochs with absolute amplitudes larger than 100 µV were rejected. The rest of the responses were averaged and smoothed by a second-order polynomial Savitzky-Golay filter across 17 samples. (The number of samples was determined empirically to remove high-frequency noise.)

The mean interpeak amplitudes (P100-(N75 + N145)/2) and the P100 peak time were evaluated offline. If the peak P100 was not identifiable, we assumed that its mean interpeak amplitude was equal to zero and the peak time was set as not available.

#### Motion-onset VEPs

To elicit motion-onset VEPs, we used a radial circular pattern corrected for equal visibility in the whole stimulus field by a magnification factor [m. factor = 1/(0.1 × eccentricity [°] + 1)] [25]. The local motion velocity increased (5–25°/s), while the spatial frequency decreased (1–0.2 cycle/°) toward the periphery. (The temporal frequency of 5.1 cycle/s was constant over the whole stimulus field.) The structure moved for 200 ms; then, it was stationary for 1000 ms. To avoid direction-specific adaptation that would result in a motion aftereffect, we changed the motion direction randomly (centrifugal or centripetal). Two variants of motion stimulation were used: central and peripheral. During the central variant, the stimulus occupied the central 8° of the screen (*M-VEP C8°*), while for the peripheral variant, the central 20° was masked by a gray circle of pattern average luminance with a fixation cross in its center (*M-VEP M20°*). The mean luminance of 17 cd/m^2^ was constant. One motion-onset VEP examination took approximately 60 s and consisted of forty stimuli. Each VEP variant was examined twice. The patients’ task was to keep their gaze on the fixation point during the recording.

As in the case of pattern-reversal VEPs, EEG poststimulus epochs of 440 ms duration were sampled at 500 Hz. Epochs with an absolute amplitude larger than 100 µV were rejected. The rest of the responses were averaged and smoothed by a second-order polynomial Savitzky-Golay filter across 47 samples.

The mean interpeak amplitudes ((P1 + P2)/2-N2) and the N2 peak time were evaluated offline. If the N2 peak was not identifiable, we assumed that its mean interpeak amplitude was equal to zero and the peak time was set as not available.

#### Cognitive ERPs

ERPs were recorded during an oddball test in which the white letter X (frequent, nontarget stimulus with a probability of 75%) and Arabic digits 1–9 (rare target stimulus with a probability of 25%) appeared pseudorandomly. The “X” or the digit of 5.7 × 6.3° was displayed for 500 ms in the center of the black stimulus field, which was followed by a blank screen with the fixation point displayed for 500 ms. The mean luminance was 1 cd/m^2^. The patients were instructed to press a handheld button as soon as possible whenever the rare stimulus appeared. This enabled evaluation of not only the peak time and amplitude of the main ERP peak P3b (designated in the following text as P300) but also the reaction time (RT). Before the examination, a short training phase took place during which the patient learned the task.

Twenty poststimulus EEG epochs of 1000 ms duration to target stimuli and 20 randomly selected epochs to nontarget stimuli were sampled at 250 Hz. Epochs with an absolute amplitude larger than 200 µV were rejected. The rest of the responses were averaged and smoothed by a second-order polynomial Savitzky-Golay filter across 17 samples. The mean interpeak amplitude (P3 − (N2 + N3)/2), the P300 peak time, and the reaction times were evaluated offline. If the peak P300 was not identifiable, we assumed that its mean interpeak amplitude was equal to zero and the peak time was set as not available.

Our stimulation parameters and conditions were already described elsewhere [9], and they were intentionally kept similar to those used in our previous study of the intraocular mirror telescope—OriLens (LMI-Lipshitz Macular Implant). The only difference between the two studies was the pattern check size. In the present study, we used the ISCEV standard (60′ and 15′) check size for the pattern-reversal VEPs.

### Statistical analyses

To assess the effect of implantation, we compared preoperative examinations of the implanted eye values to the postimplantation measurements. The extracted peak times, average amplitudes, contrast sensitivity, and visual acuities were statistically processed with R software version 3.6.2 [26] using the “nortest” and “ggplot2” packages. The VEP/ERP curves were processed using Matlab environment release 2019b for documentation.

Based on the Anderson–Darling assessment of normal data distribution, Student’s parametric or Wilcoxon nonparametric paired tests were used to compare differences between selected visits. For NVA, the Wilcoxon nonparametric paired test was used because of the ordinal type of data.

For detection of a parameter change over the follow-up period, a linear trend analysis was performed as a post hoc analysis. Every patient’s linear slope was calculated across visits with the exception of the second one immediately following implantation because the surgery caused a decrease in almost all parameters. The slope was calculated only when three or four readable responses were available. The set of slopes was tested against zero (no group slope was the null hypothesis).

A result was considered statistically significant when the probability level (*p*) was below the alpha level of 0.05. False-negative findings when examining the side effects of implantation can be threatening to patients. Therefore, to keep the type II error low, we did not correct the alpha level for multiple comparisons. For verification of the findings, we used the aforementioned post hoc test.

## Results

We screened 40 potential subjects for the study. Ten did not meet the inclusion criteria, and 16 disagreed with the study conditions. Most often they were concerned about surgery, hospitalization, and a long rehabilitation period. Fourteen patients (five males and nine females ranging in age from 63 to 87 years) with the fixed form of AMD were selected based on their clinical state, cooperation, and interest in SML implantation. Seven right and seven left eyes underwent implantation. The SML implantation procedures and postimplantation recovery were performed without any complications or adverse effects in all patients.

**Fig. 2:**
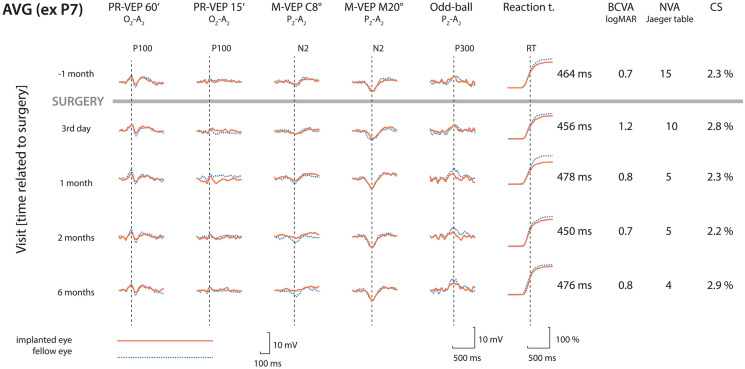
Grand average VEPs, ERPs, and the probability of a button press in the group without patient P7 and the median psychophysical parameters plotted before (above the gray line) and after (below the gray line) SML implantation. The rows represent data recorded within a single visit. The columns depict the following (from the left): months relative to the surgery; average of twice recorded VEPs from the implanted eye (red solid line) and the fellow eye (blue dashed line) elicited by the reversal of a checkerboard pattern with a check size of 60′ (labeled PR-VEP 60 ‘); reversal of a checkerboard pattern with a check size of 15 ‘ (PR-VEP15 ‘); low-contrast radial motion-onset stimuli in the central 8° (M-VEP C8°); low-contrast radial motion-onset stimuli in the periphery outside the central 20° (M-VEP M20°); target ERPs recorded in the oddball paradigm (Oddball); reaction time evaluated in response to the target stimulus (Reaction t.) depicted by the cumulative distribution function of button pressing with the median reaction time listed beside it; visual acuity (BCVA) measured on a 6-m distant high-contrast ETDRS chart and expressed as the logarithm of minimum angle (logMAR, lower number—better); near visual acuity (NVA) measured by Jaeger tables (lower number—better); and the contrast sensitivity (CS) measured by FrVACT (lower number—better). The individual results in the same format are provided in the supplementary materials

All but one patient had identifiable electrophysiological responses at least in one of the tested modalities. The only patient without reliable responses (P7–female, 87 years) had an excessive number of endogenous muscle artifacts in the recording (see Supplementary material). The behavioral responses were recorded in all patients. For the grand average curves, excluding patient P7, of VEPs, ERPs, and reaction times and the median reaction times, NVAs, BCVAs, and contrast sensitivities, see Fig. 2. For the individual results, see the accompanying supplementary material.

**Figure:**
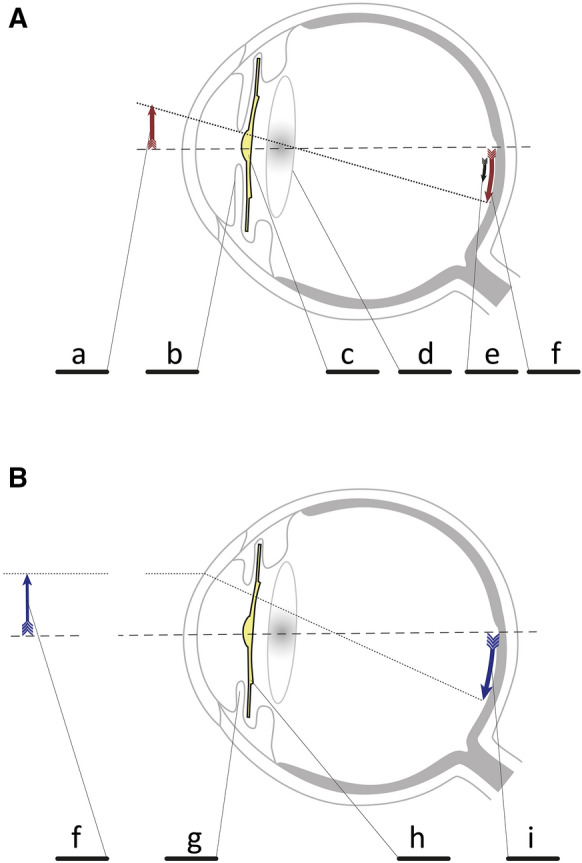
**Fig.** **1** Schematic depiction of near (panel A) and distant (panel B) vision with an implanted SML. A–An image of a close object **a** is entering the eye through a narrow pupil with a constricted iris **b**, is magnified by the central + 10 D part of the SML **c**, travels through the patient’s artificial lens, **d** and projects on the retina magnified approximately twice in size **f** of an unmagnified the image **e**. B–A distant image **f** enters the eye and projects through an opened pupil with a dilated iris **g** and the outer part of the SML **f** to be displayed on the retina without any magnification

### Pattern-reversal VEPs

In 13 out of 14 patients, we recorded the PR-VEP 60′ with a recognizable P100 component with a median peak time of 128 ms (lower quartile, 118 ms; upper quartile, 132 ms) and interpeak amplitude of 5.0 (3.6; 7.0) *µ*V before the implantation. During the follow-up period, the number of patients with a recognizable PR-VEP 60′ response oscillated between 11 and 12. Six months after the implantation, the peak time of P100 was 129 (119; 136) ms with an amplitude of 4.6 (4.1, 8.0) µV, which was not significantly different from the preimplantation examination (*n* = 11, *p* = 0.2517 vs. *n* = 14, *p* = 0.6489). Across visits, the trend for amplitudes and peak times did not reach statistical significance (p > 0.7805). We saw a significant (*n* = 11, *p* = 0.0344) decrease in the peak time to 133 (126; 137) ms at the first postimplantation visit, likely because of the impact of surgery.

**Fig. 3:**
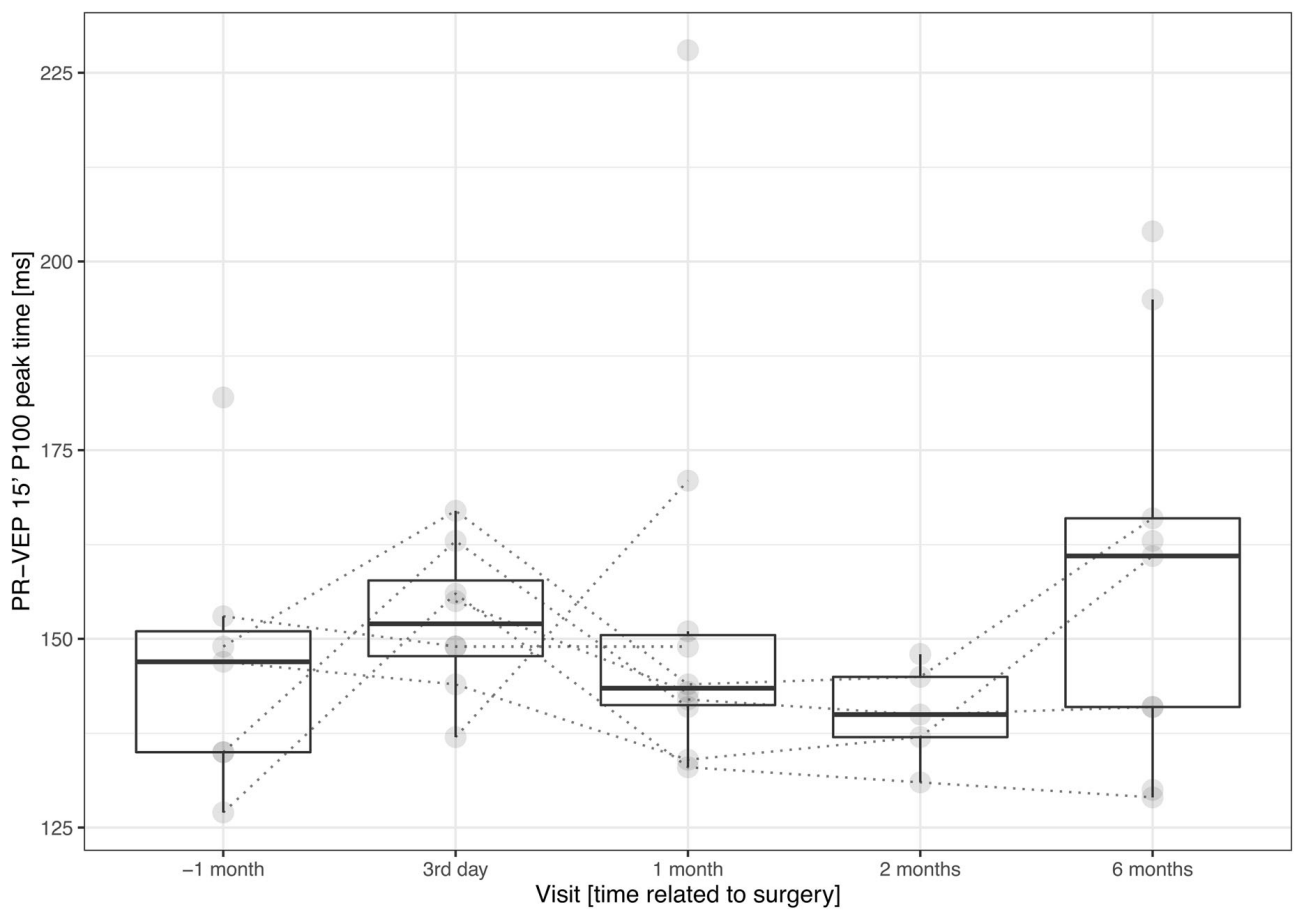
Plot combining the boxplots summarizing the development of the P100 peak time of PR-VEP 15′. The results are grouped by visit. The individual measurements are depicted as semitransparent gray points connected by dotted lines to depict within-patient relations. The plot illustrates a prolongation of the P100 peak time just after implantation and a return close to preimplantation levels within one month. With respect to the preimplantation recording, the only significant peak time prolongation was recorded during the last visit

In case of the PR-VEP 15′, we recorded a readable response in seven patients before surgery in native conditions, i.e., without any correction. The number of patients with a readable PR-VEP 15′ increased to 9 at the end of follow-up and oscillated from 5 to 10 during the follow-up period. There was a statistically significant prolongation in the P100 peak time between the preimplantation recording of 148 (138; 152) ms and the end of follow-up recording of 162 (146; 165) ms (*n* = 6, *p* = 0.0313).^2^ The mean interpeak amplitude did not change significantly (*n* = 14, *p* > 0. 3635); for particular values see Table 1 and Fig. 3. There was no statistically significant trend in the prolongation of the P100 peak time of 21 (6; 36) ms/year, (*n* = 4, *p* = 0.1250) or in the mean amplitude change of − 0.1 (− 1.4; 3.2) µV/year, (*n* = 14, *p* = 0.4318). For details, see Table 1, Fig. 2 and the supplementary material, which depict the grand averages and individual patient results.

**Table 1:** The columns (from the second one) depict time with respect to the surgery

Visit [time relative to surgery]	−1 month	3rd day	1 month	2 months	6 months
PR-VEP 60′ P100 peak time [ms] *p* = 0.7805	128 (118; 132) *n* = 13	133 (126; 136.5) * *n* = 11	130 (122; 135.5) *n* = 11	129.5 (113.5; 133) *n* = 12	129 (119; 136) *n* = 11
PR-VEP 60′ P100 amplitude [µV] *p* = 0.9862	5.0 (3.6; 7) *n* = 14	4.4 (2.8; 7.8) *n* = 14	4.4 (3.4; 6.9) *n* = 14	6.1 (2.9; 6.5) *n* = 14	4.6 (4.1; 8.0) *n* = 14
PR-VEP 15′ P100 peak time [ms] *p* = 0.1250	147 (135; 151) *n* = 7	152 (147.8; 157.8) *n* = 8	143.5 (141.2; 150.5) *n* = 10	140 (137; 145) *n* = 5	161 (141; 166) ^#^ *n* = 9
PR-VEP 15′ P100 amplitude [µV] *p *=0.4318	2 (0.9; 3.5) *n* = 14	1.9 (1.1; 4.6) *n* = 14	2.9 (0.6; 6.4) *n* = 14	0.9 (0.0; 3.9) *n* = 14	3.3 (0.5; 5.2) *n* = 14
M-VEP C8° N2 peak time [ms] *p* = 0.3125	210 (185.8; 220) *n* = 8	194 (185; 205) *n* = 8	205 (193; 226) *n* = 6	199 (193; 228) *n* = 9	211.5 (202; 219)* n* = 8
M-VEP C8° N2 amplitude [µV] *p* = 0.2240	4.1 (0.0; 6.9) *n* = 14	2.9 (0.0; 6.3) *n* = 14	2.7 (0.0; 6.4) *n* = 14	3.2 (0.0; 6.0) *n* = 14	3.2 (0.0; 5.4) *n* = 14
M-VEP M20° N2 peak time [ms] *p* = 0. 03554	191.5 (186.5; 214.5) *n* = 14	203 (192.5; 225.5) *n* = 11	191 (185; 211) *n* = 13	198.5 (188; 205.5) *n* = 12	205 (187; 218) * n = 13
M-VEP M20° N2amplitude [µV] *p* = 0.3248	6.9 (5.7; 7.9) *n* = 14	5.6 (3.9; 7) *n* = 14	6.8 (5.8; 8.5) *n* = 14	5.4 (4.6; 7.9) *n* = 14	6.6 (4.8; 7.4) *n* = 14
Odd-ball P300 peak time [ms] *p* = 0.3868	520 (506; 595) *n* = 14	508 (466; 562) *n* = 11	524 (492; 580) *n* = 13	492 (472; 560) *n* = 11	504 (470; 540) *n* = 11
Odd-ball P300 amplitude [µV] *p* = 0.9720	11.1 (7.6; 13.4) *n* = 14	10.2 (5.4; 12.9) *n* = 14	9.7 (7; 15.9) *n* = 14	11.7 (4.4; 14.4) *n* = 14	10.6 (5.2; 20.0) *n* = 13
Reaction time [ms] *p* = 0.1762	464 (425; 496) *n* = 14	456 (428; 540) *n* = 13	478 (426; 524) *n* = 14	450 (433; 542) *n* = 14	476 (442; 543) *n* = 14
BCVA [logMAR] *p* = 0.2166	0.7 (0.5; 0.8) *n* = 14	1.2 (0.8; 1.2) ^##^ *n* = 14	0.8 (0.5; 1) *n* = 14	0.7 (0.5; 0.8) *n* = 14	0.8 (0.7; 0.8) * n = 14
NVA [Jaeger table] *p* < 0.0001	J15 (J13; J17) *n* = 14	J10 (J6; J12) ^##^ *n* = 14	J5 (J3; J7) ^##^ *n* = 14	J5 (J1; J6) ^##^ *n* = 14	J4 (J1; J6) ^##^ *n* = 14
NVA +6 D before surg. [Jaeger table] *p* = 0.03667	J6 (J3; J7) *n* = 14	J10 (J6; J12) ^##^ *n* = 14	J5 (J3; J7) *n* = 14	J5 (J1; J6) *n* = 14	J4 (J1; J6) *n* = 14
CS [%] p = 0.09057617	2.3 (1.7; 2.9) *n* = 14	2.8 (2.1; 3.2) *n* = 14	2.3 (2.1; 3.2) *n* = 14	2.2 (1.8; 3.4) *n* = 14	2.9 (2.1; 4.8) *n* = 14

The rows represent the following parameters. The names for the electrophysiological parameters and reaction time are self-explanatory. For the psychophysical parameters, BCVA stands for the best-corrected visual acuity at 6 m measured by the high-contrast ETDRS chart and expressed as the logarithm of the minimum angle resolution (logMAR, lower number—better); NVA is the near visual acuity measured by Jaeger tables (lower number—better); NVA + 6 D is the NVA tested with + 6 D lens before implantation; and CS is the Michelson contrast sensitivity measured by FrVACT (lower number—better). The p-value in this column corresponds to the linear trend hypothesis test (see Methods)

### Motion-onset VEPs

For central radial motion-onset stimulation, we recorded readable M-VEP C8° from eight patients preoperatively with an N2 peak time of 210 (186; 220) ms and an interpeak amplitude of 4.1 (0.0; 6.9) *µ*V. At the end of the follow-up period, the peak time was 212 (202; 219) ms, and the amplitude was 3.2 (0.0; 5.4) µV; in the paired test, these did not reach statistical significance (*n* = 7, *p* = 0.0781 and *n* = 14, *p* = 0.6053, respectively). For other visits, we also did not find statistically significant differences compared with preimplantation values (*p* > 0.3081). We did not observe a significant linear intervisit dependence for the peak time or amplitude (*n* = 5, *p* = 0. 3125, and *n* = 14, *p* = 0.2240 respectively).

**Fig. 4:**
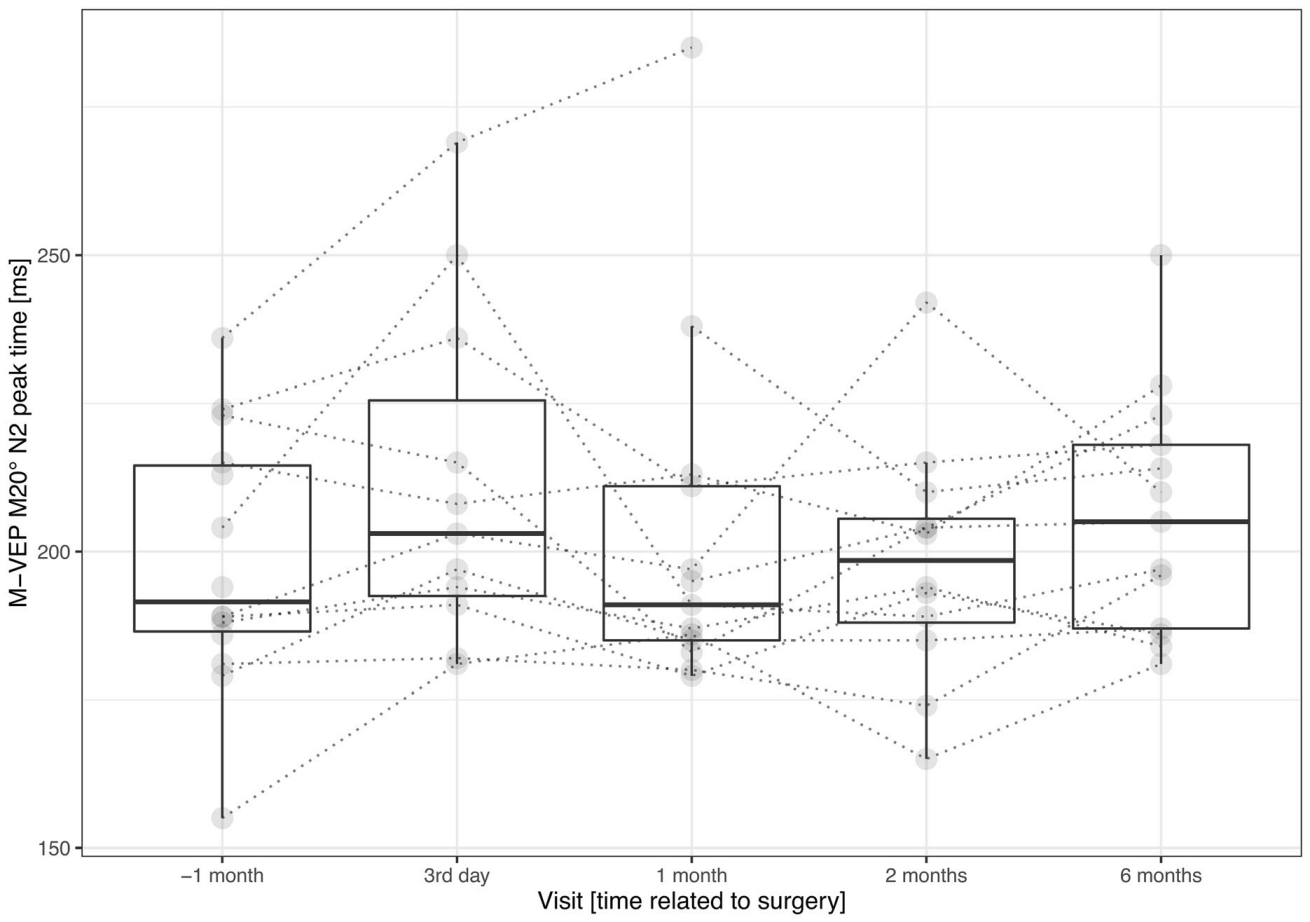
N2 peak time in peripheral radial motion-onset VEPs grouped by visit. The individual measurements are depicted as semitransparent gray points connected by dotted lines to depict within-patient relations. The plot illustrates two statistically significant findings—the paired difference between preimplantation and the first postimplantation visit (*n* = 11, *p* = 0.0284) or last visit (*n* = 13, *p* = 0.0341)

The only significant motion-onset marker was the N2 peak time of M-VEP M20°. At the preimplantation visit, we identified the N2 peak time for all patients of 192 (187; 215) ms. In the paired comparison, it was statistically significantly (*n* = 13, *p* = 0.0341) shorter by 8 (− 3; 15) ms than the N2 peak time of 205 (187; 218) ms recorded at the last visit. The mean interpeak preimplantation amplitude of 6.9 (5.7; 7.9) *µ*V was not different (*n* = 14, *p* = 0. 4420) from the last postimplantation visit value of 6.6 (4.8; 7.5) *µ*V. The trend of the N2 peak time prolongation of 14 (− 5; 27) ms/year was statistically significant (*n* = 12, *p* = 0.0355); for the amplitude, we did not see such dependency (*n* = 14, *p* = 0.3248). Similar to PR-VEP 60′, we saw significant (*n* = 11, *p* = 0.0284) N2 peak time prolongation to 203 (193, 226) ms at the first postimplantation visit. For details, see Table 1 and Fig. 4.

### Odd-ball ERPs

At the preimplantation visit, we recorded a distinct P300 wave for all patients. The P300 peak time of 520 (506; 595) ms and its amplitude of 11.1 (7.6; 13.4) µV were not significantly different (*n* = 11, *p* = 0.4136 and *n* = 13, *p* = 0.9251) from the peak time and amplitude values of 504 (470; 540) ms and 10.6 (5.2; 20.0) µV, respectively, recorded during the last visit. In addition, for the reaction time, we did not observe significant difference (*p* = 0.1245) between the preimplantation value of 464 (425; 496) ms and the last follow-up measurement of 476 (442; 543) ms. For these parameters, there were not significant differences compared with other visits (*p* > 0.1092), and the trend analysis did not show a significant slope (*p* > 0.1762).

### Distant visual acuity

The median BCVA before surgery was 0.7 (0.5; 0.8) logMAR. Three days after implantation, we observed a statistically significant (*n* = 14, *p* = 0.0041) decrease in the BCVA to 1.2 (0.8; 1.2) logMAR. In the following records, we saw a return to the preimplantation level, and two months after the surgery, with a value of 0.7 (0.5; 0.7) logMAR, there was not statistically significant difference (*n* = 14, *p* = 0.8627). The last BCVA of 0.8 (0.7; 0.8) logMAR that was measured six months after implantation worsened significantly (*n* = 14, *p* = 0.0132) compared to the preimplantation level. The development of the BCVA is summarized in Table 1. The slope analysis did not show any trend (*n* = 14, *p* = 0.2166).

### Near visual acuity

**Fig. 5:**
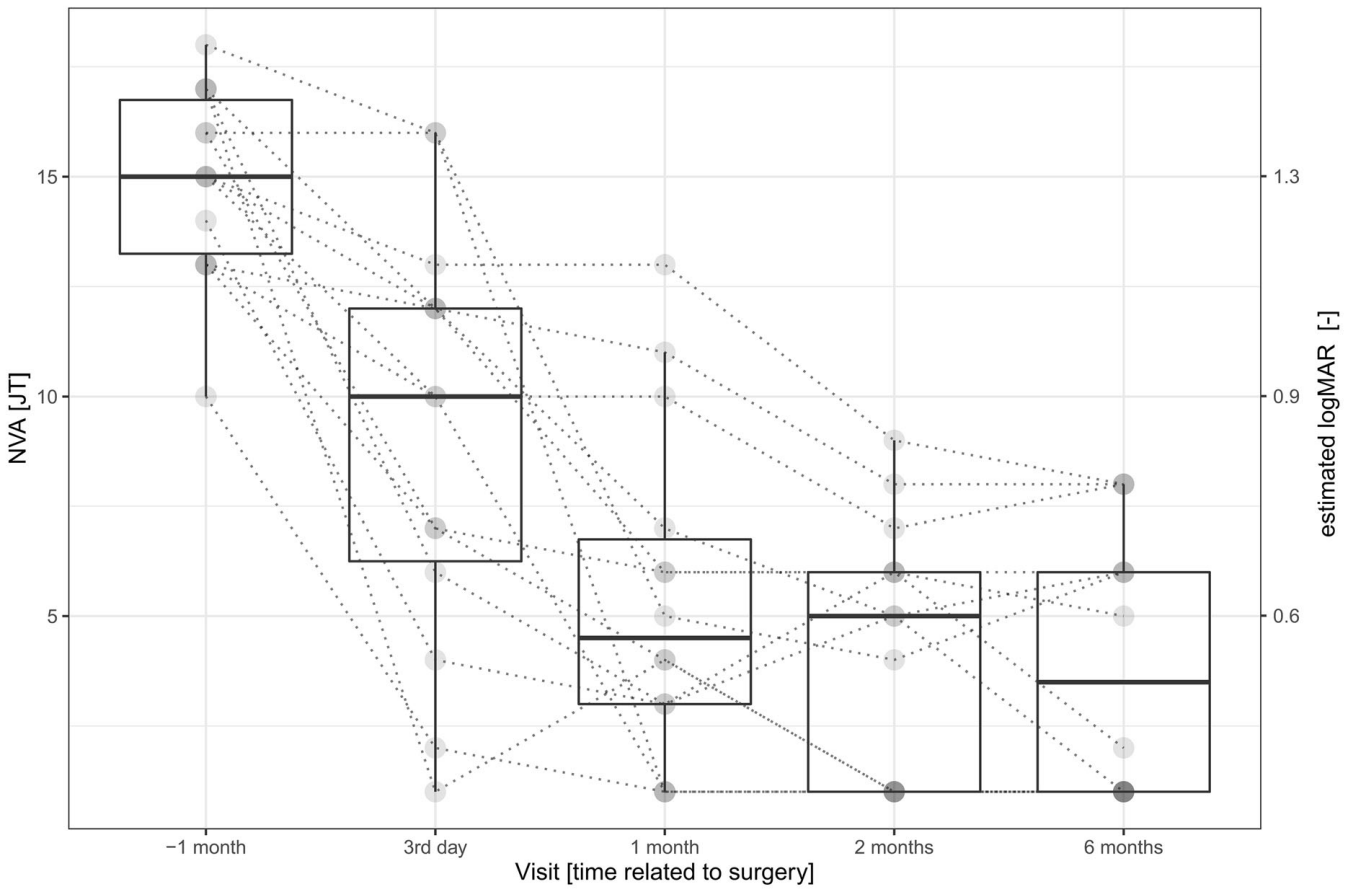
Near visual acuity measured without any correction grouped by visit. The individual measurements are depicted as semitransparent gray points connected by dotted lines to depict within-patient relations. The plot illustrates the continuous improvement in near visual acuity after the implantation

Preoperatively, we measured the NVA of J15 (J13, J17) for a native condition and J6 (J3, J7) with + 6 D lenses. Postoperatively, only the native condition was examined, and the NVA was J4 (J1; J6) at the end of the follow-up period. Postoperatively, all NVA measurements were significantly decreased compared with the native preimplantation condition (*n* = 14, *p* < 0.0016). The slope analysis showed a significant (*p* < 0.0001) decrease in the Jaeger table number (increase in the NVA) during the follow-up period. Compared with preoperative + 6 D condition, we found a significantly (*n* = 14, *p* = 0.0075) worse NVA J10 (J6, J9) on the third day postoperatively, which improved and was not significantly different (*p* > 0.1340) during the rest of the follow-up period. For details, see Table 1, Fig. 5, and the supplementary material.

### Contrast sensitivity

Viewed natively before implantation, the lowest contrast allowing discrimination of the 480′ Landolt C orientation was 2.3 (1.7; 2.9)%. This was not significantly different (*n* = 14, *p* > 0.0785) from the postimplantation contrast sensitivity 2.9 (2.1; 4.8)%. The trend analysis did not reject the hypothesis that the slope is different from zero (*n* = 14, *p* = 0.0906). For details, see Table 1 and the supplementary material.

The cells from the second row and the second column contain descriptive statistics, the median (upper, lower quartile), and the number of observations (n). In cases in which the measurement was significantly different from the preimplantation value, the stars and hashes indicate Student’s or Wilcoxon (respectively) paired tests of significance: * or ^#^ corresponds to *p* < 0.05 and *p ≥ *0.01, ** or ^##^ corresponds to *p* < 0.01 and *p ≥ *0.001, and *** or ^###^ corresponds to *p* < 0.001. When a VEP peak was unidentifiable, we assigned 0 μV to the corresponding amplitude, and the peak time was set as not available; therefore, the number of observations might differ for the peak time and amplitude.

## Discussion

Using electrophysiological examination in this prospective study, we followed fourteen patients before and 6 months after SML implantation to evaluate effect of implantation on their visual processing and to assess whether motion-onset VEPs are suitable to monitor the vision of patients with AMD.

A significant effect of implantation on VEPs was already present during the first postoperative measurement. Although patients’ near visual acuity improved, we observed a stronger transient deterioration in distant vision and a prolongation of the peak time to pattern-reversal of the checkerboard 60′ and the motion-onset stimuli in the peripheral field.

In the long term, the continuous and robust improvement in near visual acuity was accompanied by a slight but significant N2 peak time prolongation of peripheral motion-onset VEPs during follow-up and by a decrease in the P100 peak time of PR-VEP 15′ at the last visit. Such VEP deterioration was supplemented by a small decrease in distant visual acuity. The other electrophysiological results did not show any significant change.

Since the development of the SML principle and its use in maculopathies [10], four papers evaluating the effect of SML [11–13, 27] and one case report of a patient with diabetic macular disease [28] have been published (pubmed.gov searched for “Scharioth macula lens” in July 2020). The studies agreed that the SML improves near visual acuity and does not affect distant vision. None of these works used visual-evoked potentials.

Regarding the use of electrophysiological examination of patients with AMD and an optical implant, we found only one published report (pubmed.gov searched for “visual evoked potential macular degeneration implant” in June 2020) describing the longitudinal monitoring of a 70-year-old female and a 90-year-old male with LMI implants [9]. While distant visual acuity increased after LMI implantation, and peripheral projection was preserved, the M-VEP M20° peak time was prolonged after implantation. The authors showed that electrophysiological examination may help reveal a neglected factor of light attenuation caused by LMI. Based on the VEP results, they tested contrast sensitivity, which showed a postimplantation decrease [9]. These results led us to measure the contrast sensitivity, which did not change significantly after SML implantation.

For the sensitivity of pattern-reversal in AMD patients, there are studies reporting a decrease in the amplitude and a prolongation of the peak time of the P100 wave [29–32]. We compared our patients with respect to the laboratory norm of the P100 peak time recorded at a high spatial frequency structure reversal. The P100 wave was missing in seven patients or had a peak time longer than 141 ms in four patients. The incidence of abnormality was 79%, which is exactly the same as in the study by Niermann et al. [31]. This is not surprising as the VEPs for high spatial frequency pattern-reversal depend mostly on foveal representation [33].

Other than the mentioned case study of LMI, no studies have evaluated motion-onset VEPs in AMD patients (pubmed.gov searched for “motion-onset visual evoked potential macular degeneration” in July 2020). In our study, responses to central motion-onset stimulation were affected similarly to pattern-reversal VEPs; the N2 peak was missing in six patients and prolonged above 211 ms in four patients. The situation was different for stimulation in the periphery of the visual field. In the preimplantation period, nine patients had a dominant peak within the normal limit (below 212 ms), and the remaining five exceeded the norm by 1, 3, 11, 12, and 24 ms. The number of normal findings might be underestimated, however, since the norm was created for controls aged 50–60 years [34]. A similar preservation of electrophysiological responses to visual motion was described for amblyopia; the authors found significantly reduced responses to pattern-reversal stimulation, while responses to the motion-onset stimulation were comparable to the control group [35]. In these two disorders, the common electrophysiological outcome is caused by reduced central and preserved peripheral vision of different etiologies, which is consistent with the low specificity of VEPs.

With many unrecordable reactions in AMD, the high sensitivity of PR-VEP does not seem to be enough for monitoring vision changes in these patients. For this purpose, peripheral low-contrast motion-onset stimulation is suitable to monitor for comorbidities or changes in the visual system because M-VEP M20° was detectable in most of our AMD patients, and, as a concurrent necessary condition, it is sufficiently sensitive in various diseases such as neuritis, multiple sclerosis, and neuroborreliosis (for review see [15]).

Our study supported a generally accepted view that near visual acuity, measured without any additional correction, improves significantly after SML implantation [12, 13, 27]; these studies also claim that distant visual acuity remains without a statistical change; however, there was an observable minor decrease across these studies.^3^ We found that the BCVA underwent statistically significant changes. After implantation, the BCVA decreased significantly, returned to preimplantation level after a month, and then decreased again after 6 months (see Table 1). To support the view that distant vision is undergoing development, one should consider the parallel changes we measured in response to peripheral motion-onset stimulation. They were also reflected by trend analysis, which was not statistically significant for BCVA likely because of higher intervisit dynamics (see the supplementary material). Despite the statistical significance, just one line of the logMAR chart differentiated the preimplantation from the last postimplantation visit BCVA.

The change in distant and peripheral vision could be due to the optical properties of the SML or brain adaptation to a new monocular situation. A change in the optical properties of one eye to enhance near vision creates so-called monovision, which might modify VEPs [36]. Since the change in VEPs was slow, its origin resembles brain plasticity in response to monovision rather than direct optical degradation of distant and peripheral images by the SML. Aging as another reason for the changes in distant and peripheral vision [37] is unlikely because the M-VEP M20° N2 peak time has a trend of 0.8 ms/year in the normal population between 18 and 60 years [34], and we measured a decrease of 14 ms/year in present study. Furthermore, in contrast to the results in the implanted eyes, in the fellow eyes, we did not see a significant difference (*n* = 11, *p* = 0.7555) in the M-VEP M20° N2 peak time between preimplantation and six months postimplantation.

Thus, we conclude that in addition to the strong and desirable effect on near vision, there is a long-term consequence of SML implantation in a small decrease in distant and peripheral vision in the implanted eye.

In our study, we found a seeming dissociation between psychophysical and electrophysiological recordings. The reason the electrophysiological results did not follow the NVA improvement could be due to the different observation distance. It was approximately 15 cm for the NVA and 60 cm for the electrophysiological examinations, which was out of the SML focus (15 cm). In addition, during the NVA test, we used the subject’s desired illumination, which was usually higher than normal daylight, which, in combination with the accommodation convergence reflex for close viewing distance, reduced possible corneal image spreading and contributed to the better NVA.

## Conclusions

We proved that measurement of VEPs can be used to evaluate the vision of patients with AMD and to monitor changes related to monovision.

With an observation distance of 60 cm, the electrophysiological markers did not reach or surmount the preimplantation level, while the near visual acuity without an external aid improved strongly. Electrophysiological testing brought valuable outcomes in evaluating distant and peripheral vision.

Using motion-onset peripheral stimulation, we recorded a canonical form of motion-onset VEPs in the majority of our AMD patients throughout the follow-up period. This helped to reveal a long-term consequence of SML implantation as a decrease in distant and peripheral vision in the implanted eye. This effect is negligible compared with the near vision enhancement. Pattern-reversal VEPs were also a sensitive test but only partially effective as they were not recordable in many patients.

## Limitations

The electrophysiological examinations were influenced by patients’ reduced ability to fixate due to maculopathy. Therefore, some recordings have a lower S/N ratio, which might decrease the sensitivity of the testing. We tried to reduce this effect using a bigger fixation cross of 1° and by including a peripheral motion stimulus.

In the simple oddball discrimination task, it is not clear how much a learning during repeated examinations might confound the cognitive function evaluation.

The specific effect of the SML was not evident in our VEP examination; however, testing with a very close viewing distance (15 cm) could be an appropriate condition for its evaluation. Our results are indicative of routine VEP examinations. Also, CS measure was examined from the same distance having the same limitations.

Our monitored electrophysiological parameters alone are not sufficient to estimate the effectiveness of treatment with the SML. For such conclusions, it is necessary to evaluate the quality of life of patients, which is included in the continuation of our project.

## Supplementary information

Supplementary file 1(PDF 2,451 kb)

Supplementary file 2 (PDF 493 kb)

## Data Availability

The dataset is obtainable from the corresponding author on reasonable request.

## Abbreviations

SML: “Scharioth macula lens”-intraocular reading lens
LMI: Lipshitz macular implant-intraocular mirror telescope OriLens
AMD: Age-related macular degeneration
CNV: Choroidal neovascularization
VEP: Visual-evoked potential
ERP: Event-related potential
PR-VEP: Pattern-reversal VEP
PR-VEP 60′: PR-VEP evoked by checkerboard of 60′ checks
PR-VEP 15′: PR-VEP evoked by checkerboard of 15′ checks
M-VEP: Motion-onset VEP
M-VEP C8°: M-VEP evoked by central 8° stimulus
M-VEP M20°: M-VEP evoked by peripheral stimulus outside central 20°
NVA: Near visual acuity
BCVA: Best-corrected distant visual acuity
ETDRS: Early treatment diabetic retinopathy study
P100, N75, N145: Peak names of the PR-VEP
P1, N2, P2: Peak names of the M-VEP
P3b, P300: Peak names of ERP

## Footnotes

1 The logMAR we counted as the angle of 1/5 of the height of the letter ***a***.

2 Note that the descriptive parameters of the peak time for individual visits (Table 1) may not always match those in pairwise comparisons. This is due to a decrease in paired values entered in the test because of missing observations.

3 Nekolova et al. [12] described a BCVA before implantation of 0.57 (min 0.5, max 0.7) logMAR and 0.62 (min 0.5, max 0.7) logMAR after six months for 8 eyes. For the BCVA measured in 50 eyes, Shrinivashan et al. [13] did not find any difference before (0.7 ± 0.9 logMAR) and after one year (0.7 ± 1.1 logMAR); however, for the uncorrected distant visual acuity, they measured a value of 0.7 ± 1.1 logMAR before implantation vs. 0.8 ± 1.1 logMAR one year later. In 15 eyes, Bereczki et al. [27] described a value of 0.34 ± 0.92 logMAR before implantation and 0.37 ± 0.92 logMAR three months later.

## Code availability

The analysis script is available from the corresponding author on reasonable request.
