# Supplementary material for: Vision before and after scharioth macular lens implantation in patients with AMD: an electrophysiological study
Source: Doc Ophthalmol. 2021 Jan 3;143(1):17–31. doi: 10.1007/s10633-020-09814-8 (PMC8266777; doi:10.1007/s10633-020-09814-8)
Supplement: Supplementary file 1 — (PDF 2,451 kb) [file 10633_2020_9814_MOESM1_ESM.pdf]

Vision before and after Scharioth macular lens implantation in patients with AMD: an electrophysiological study

Kremláček Jan, Nekolová Jana, Středová Markéta, Langrová Jana, Szanyi Jana, Kuba Miroslav, Kubová Zuzana, Vít František, Voda Petr, Veselá Martina, Jirásková Naďa

Supplementary material part A

Individual electrophysiological and psychophysical results

Electrophysiological results and psychophysical parameters before (above the gray line) and after (below the gray line) SML implantation in a single subject. The rows represent data recorded within a single visit. The columns depict the following(from the left): months relative to the surgery; twice recorded VEPs from the implanted eye (black solid line) and the fellow eye (gray dashed line) elicited by the reversal of a checkerboard pattern with a check size of 60' (labeled PR-VEP 60');reversal of a checkerboard pattern with a check size of 15' (PR-VEP15'); low-contrast radial motion-onset stimuli in the central 8° (M-VEP C8°); low-contrast radial motion-onset stimuli in the periphery outside the central 20° (M-VEP M20°); target ERPs recorded in the oddball paradigm (Oddball) with only one ERP recording; reaction time evaluated in response to the target stimulus (Reaction t.) depicted by the cumulative distribution function of button pressing with the median reaction time listed beside it; visual acuity (BCVA) measured on a 6-m distant high-contrast ETDRS chart expressed as the logarithm of the minimum angle resolution (logMAR, lower number—better); near visual acuity (NVA) measured by Jaeger tables (lower number—better); and contrast sensitivity (CS) measured by FrVACT (lower number—better).

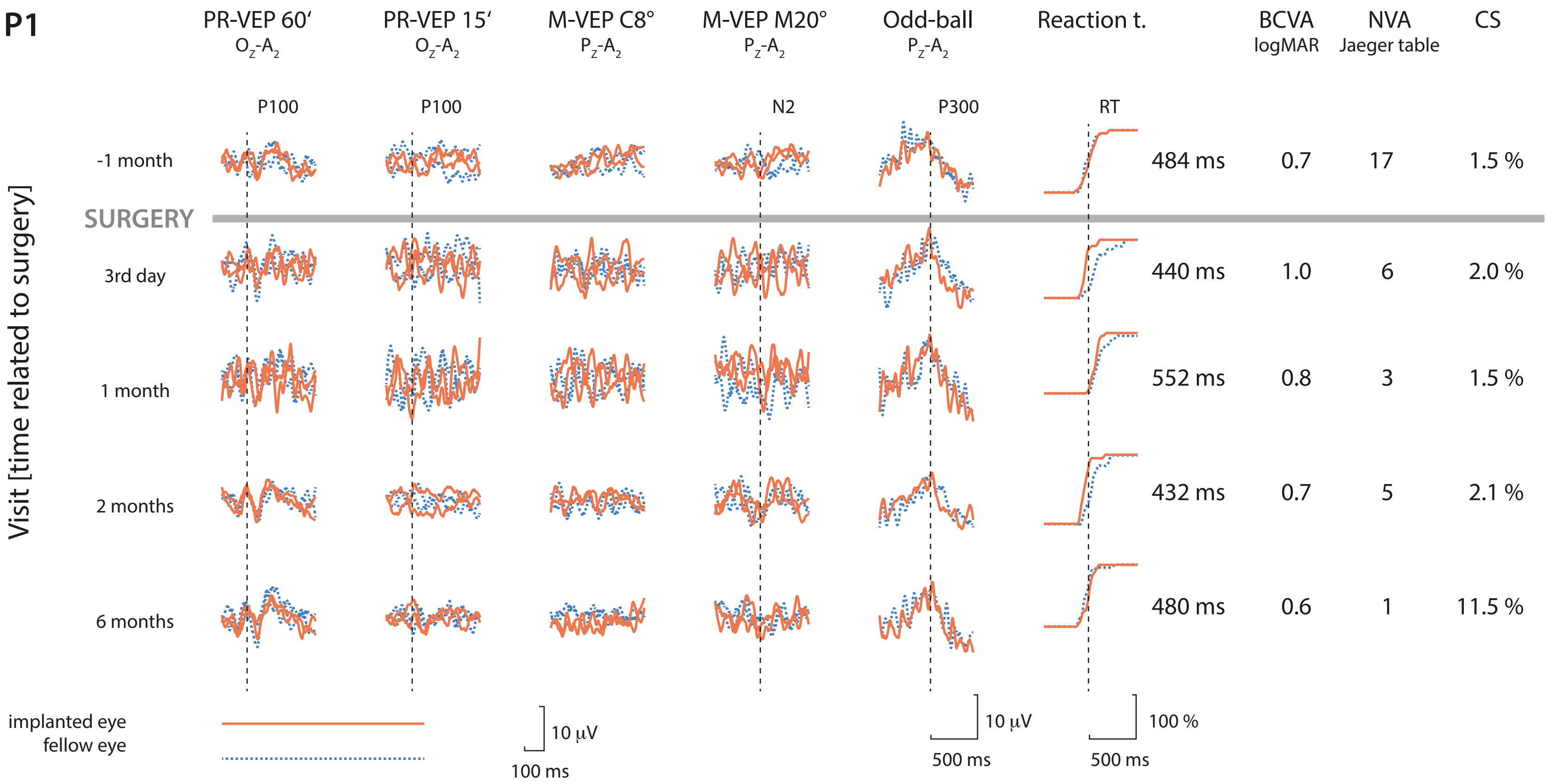

P1
PR-VEP 60'
O_Z-A_2
PR-VEP 15'
O_Z-A_2
M-VEP C8°
P_Z-A_2
M-VEP M20°
P_Z-A_2
Odd-ball
P_Z-A_2
Reaction t.
BCVA
logMAR
NVA
Jaeger table
CS
P100
P100
N2
P300
RT
Visit [time related to surgery]
-1 month
484 ms
0.7
17
1.5 %
SURGERY
3rd day
440 ms
1.0
6
2.0 %
1 month
552 ms
0.8
3
1.5 %
2 months
432 ms
0.7
5
2.1 %
6 months
480 ms
0.6
1
11.5 %
implanted eye
fellow eye
10 μV
100 ms
10 μV
500 ms
100 %
500 ms

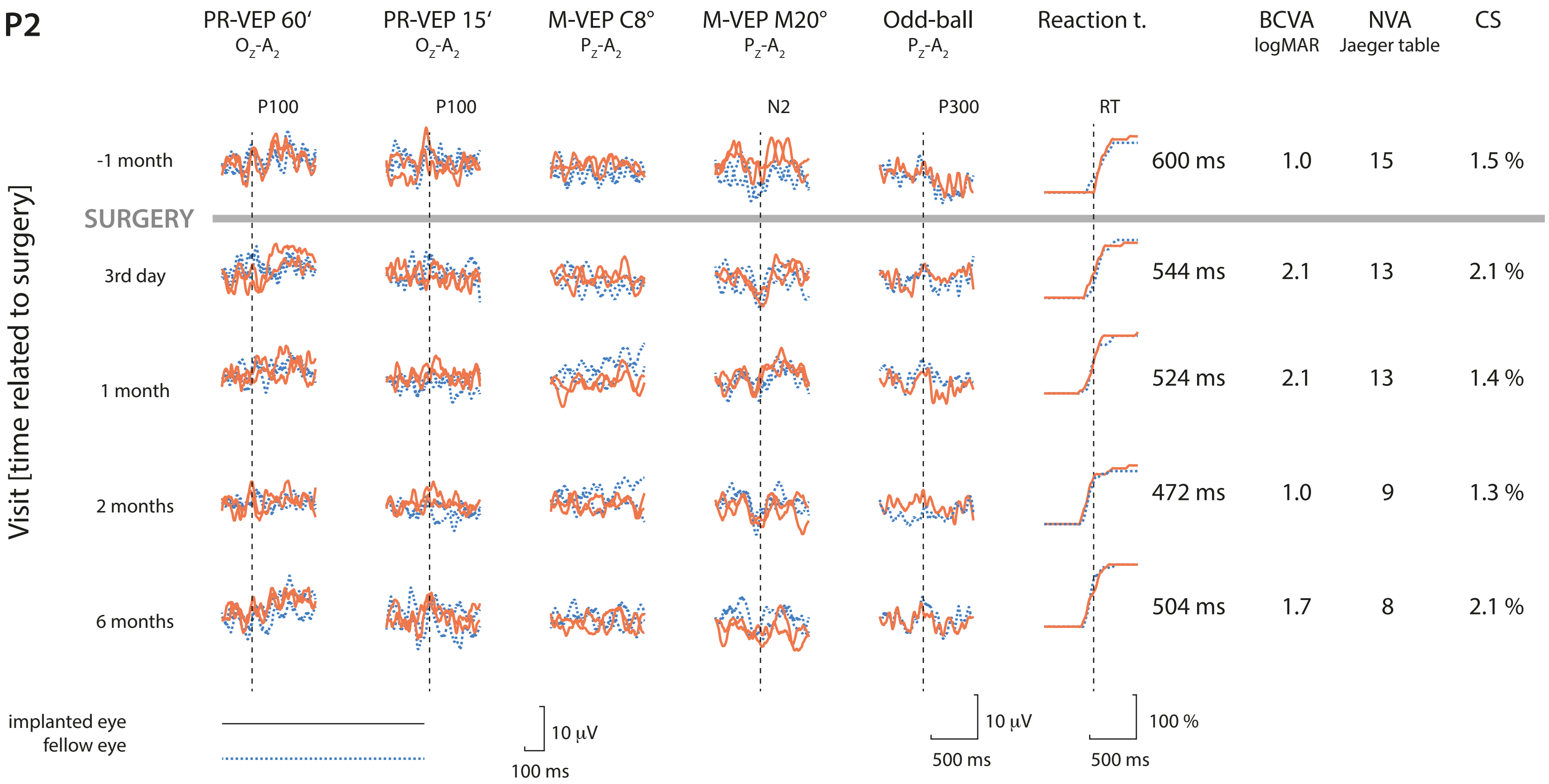
P2
PR-VEP 60‘
O_Z-A_2
PR-VEP 15‘
O_Z-A_2
M-VEP C8°
P_Z-A_2
M-VEP M20°
P_Z-A_2
Odd-ball
P_Z-A_2
Reaction t.
BCVA
logMAR
NVA
Jaeger table
CS
P100
P100
N2
P300
RT
Visit [time related to surgery]
-1 month
600 ms
1.0
15
1.5 %
SURGERY
3rd day
544 ms
2.1
13
2.1 %
1 month
524 ms
2.1
13
1.4 %
2 months
472 ms
1.0
9
1.3 %
6 months
504 ms
1.7
8
2.1 %
implanted eye
fellow eye
10 μV
100 ms
10 μV
500 ms
100 %
500 ms

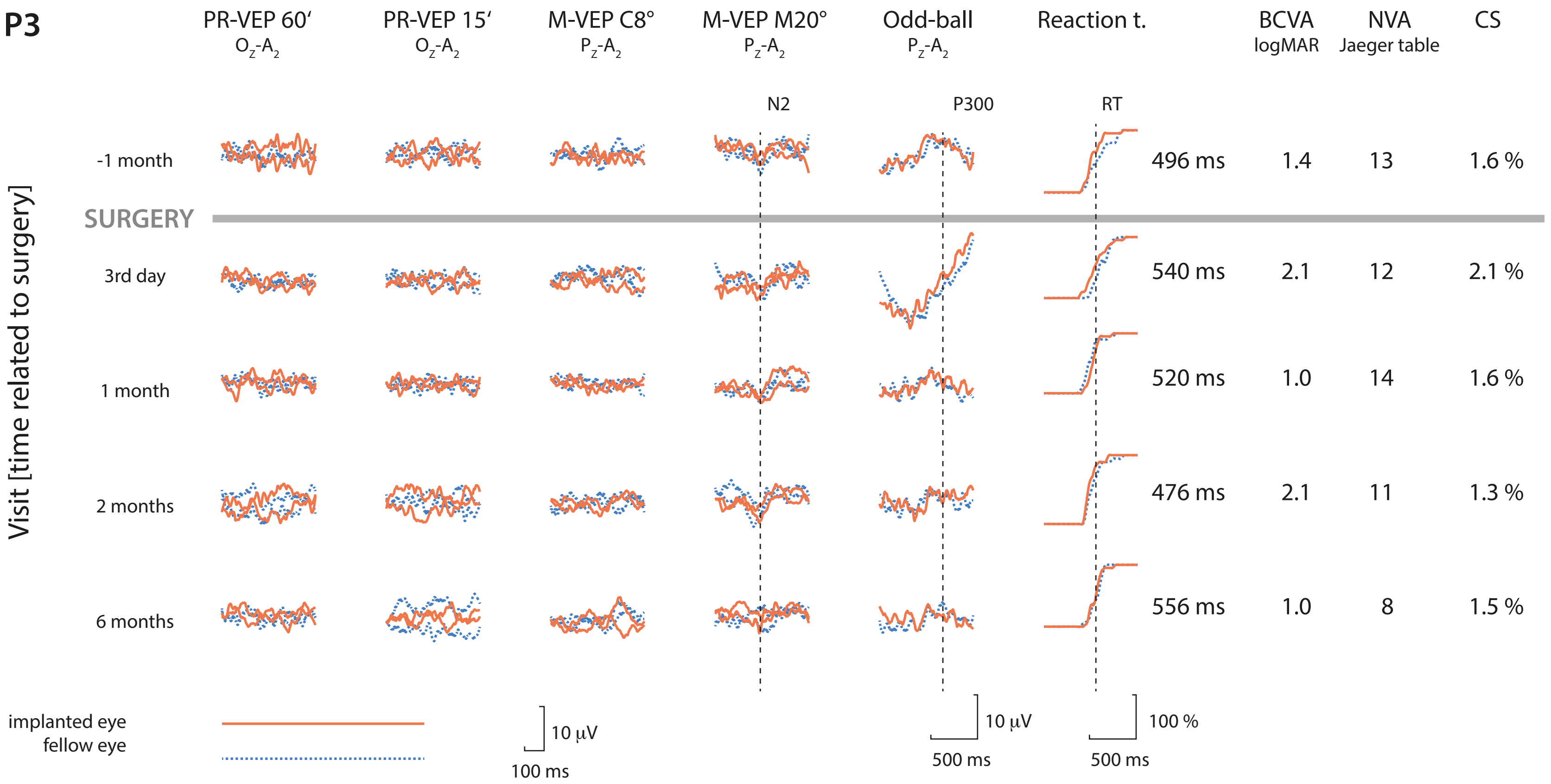

P3
PR-VEP 60'
Oz-A2
PR-VEP 15'
Oz-A2
M-VEP C8°
Pz-A2
M-VEP M20°
Pz-A2
Odd-ball
Pz-A2
Reaction t.
BCVA
logMAR
NVA
Jaeger table
CS
N2
P300
RT
Visit [time related to surgery]
-1 month
496 ms
1.4
13
1.6 %
SURGERY
3rd day
540 ms
2.1
12
2.1 %
1 month
520 ms
1.0
14
1.6 %
2 months
476 ms
2.1
11
1.3 %
6 months
556 ms
1.0
8
1.5 %
implanted eye
fellow eye
10 µV
100 ms
10 µV
500 ms
100 %
500 ms

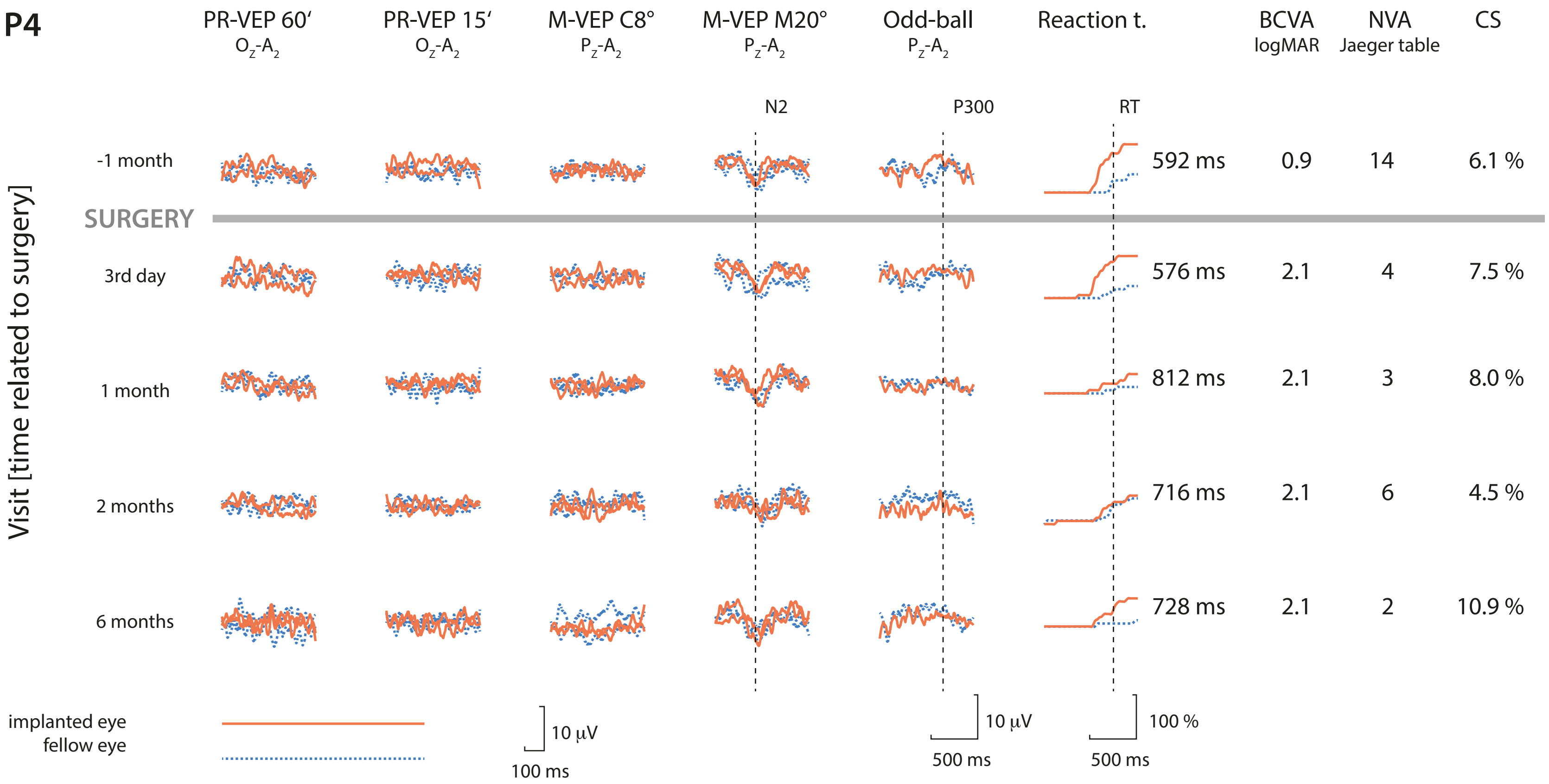
P4
PR-VEP 60‘
O_Z-A_2
PR-VEP 15‘
O_Z-A_2
M-VEP C8°
P_Z-A_2
M-VEP M20°
P_Z-A_2
Odd-ball
P_Z-A_2
Reaction t.
BCVA
logMAR
NVA
Jaeger table
CS
N2
P300
RT
Visit [time related to surgery]
-1 month
592 ms
0.9
14
6.1 %
SURGERY
3rd day
576 ms
2.1
4
7.5 %
1 month
812 ms
2.1
3
8.0 %
2 months
716 ms
2.1
6
4.5 %
6 months
728 ms
2.1
2
10.9 %
implanted eye
fellow eye
10 μV
100 ms
10 μV
500 ms
100 %
500 ms

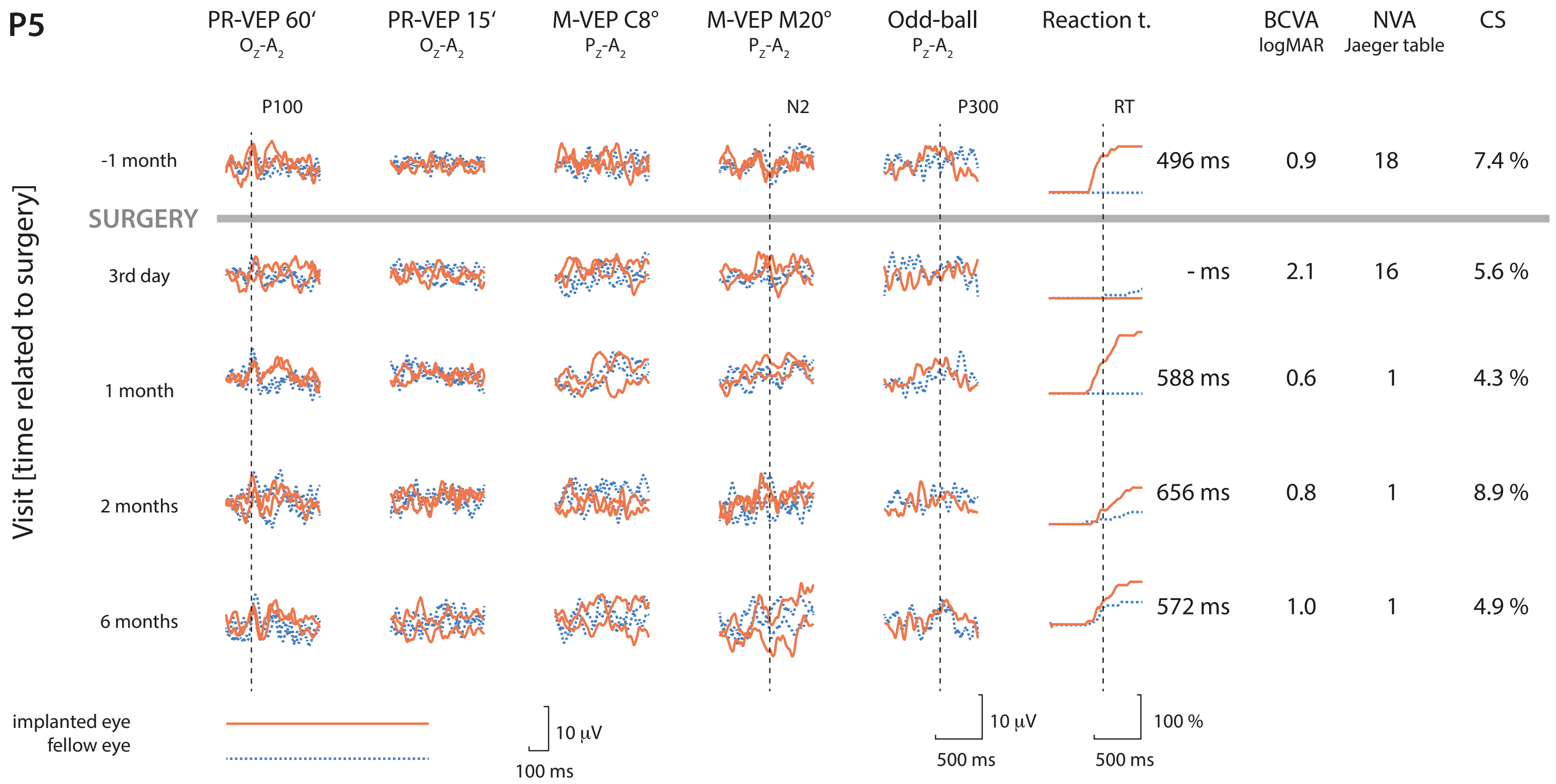

P5
PR-VEP 60'
O_Z-A_2
PR-VEP 15'
O_Z-A_2
M-VEP C8°
P_Z-A_2
M-VEP M20°
P_Z-A_2
Odd-ball
P_Z-A_2
Reaction t.
BCVA
logMAR
NVA
Jaeger table
CS
P100
N2
P300
RT
-1 month
496 ms
0.9
18
7.4 %
SURGERY
3rd day
- ms
2.1
16
5.6 %
1 month
588 ms
0.6
1
4.3 %
2 months
656 ms
0.8
1
8.9 %
6 months
572 ms
1.0
1
4.9 %
Visit [time related to surgery]
implanted eye
fellow eye
10 μV
100 ms
10 μV
500 ms
100 %
500 ms

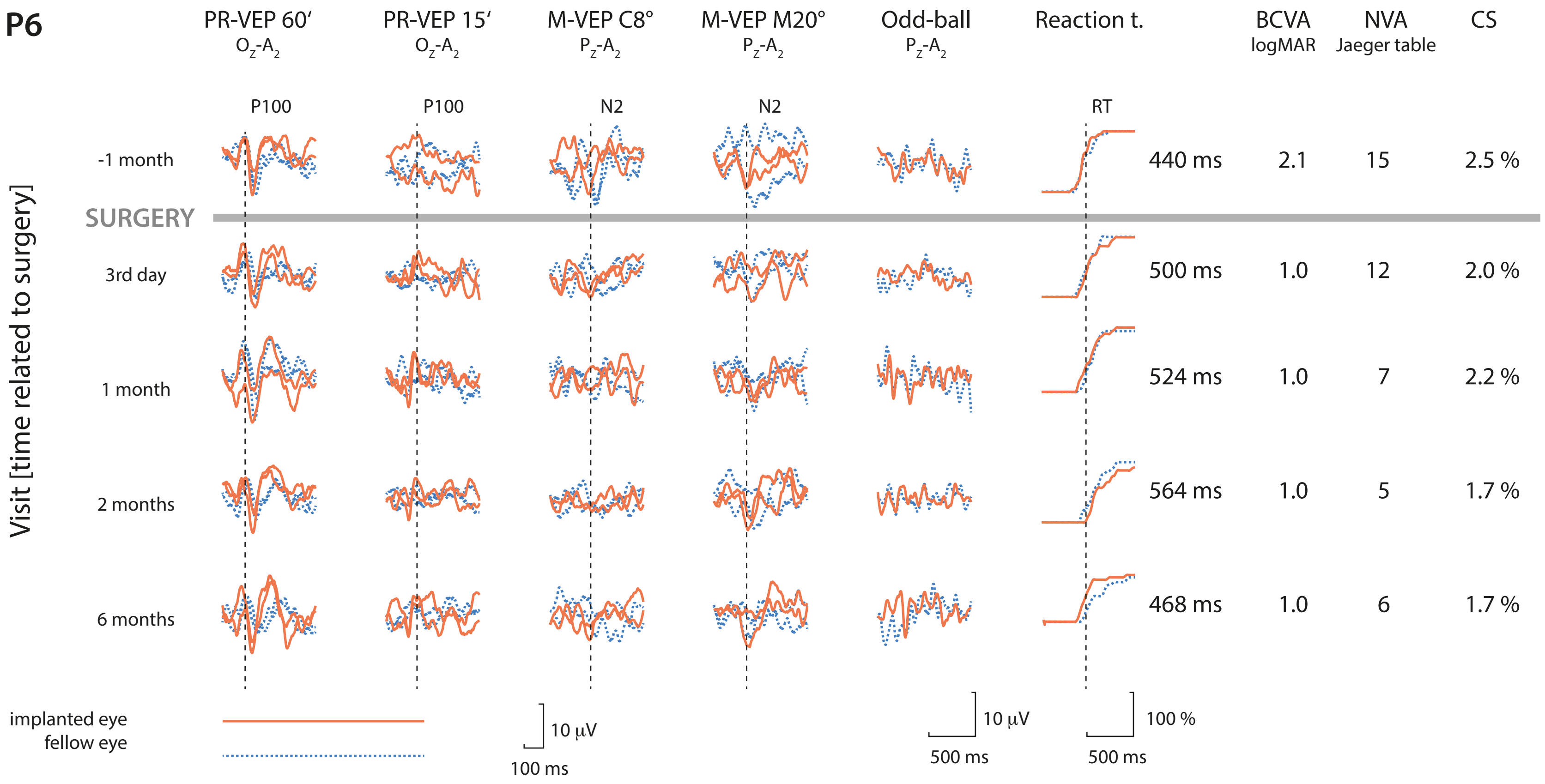

P6
PR-VEP 60‘
Oz-A2
PR-VEP 15‘
Oz-A2
M-VEP C8°
Pz-A2
M-VEP M20°
Pz-A2
Odd-ball
Pz-A2
Reaction t.
BCVA
logMAR
NVA
Jaeger table
CS
P100
P100
N2
N2
RT
Visit [time related to surgery]
-1 month
440 ms
2.1
15
2.5 %
SURGERY
3rd day
500 ms
1.0
12
2.0 %
1 month
524 ms
1.0
7
2.2 %
2 months
564 ms
1.0
5
1.7 %
6 months
468 ms
1.0
6
1.7 %
implanted eye
fellow eye
10 µV
100 ms
10 µV
500 ms
100 %
500 ms

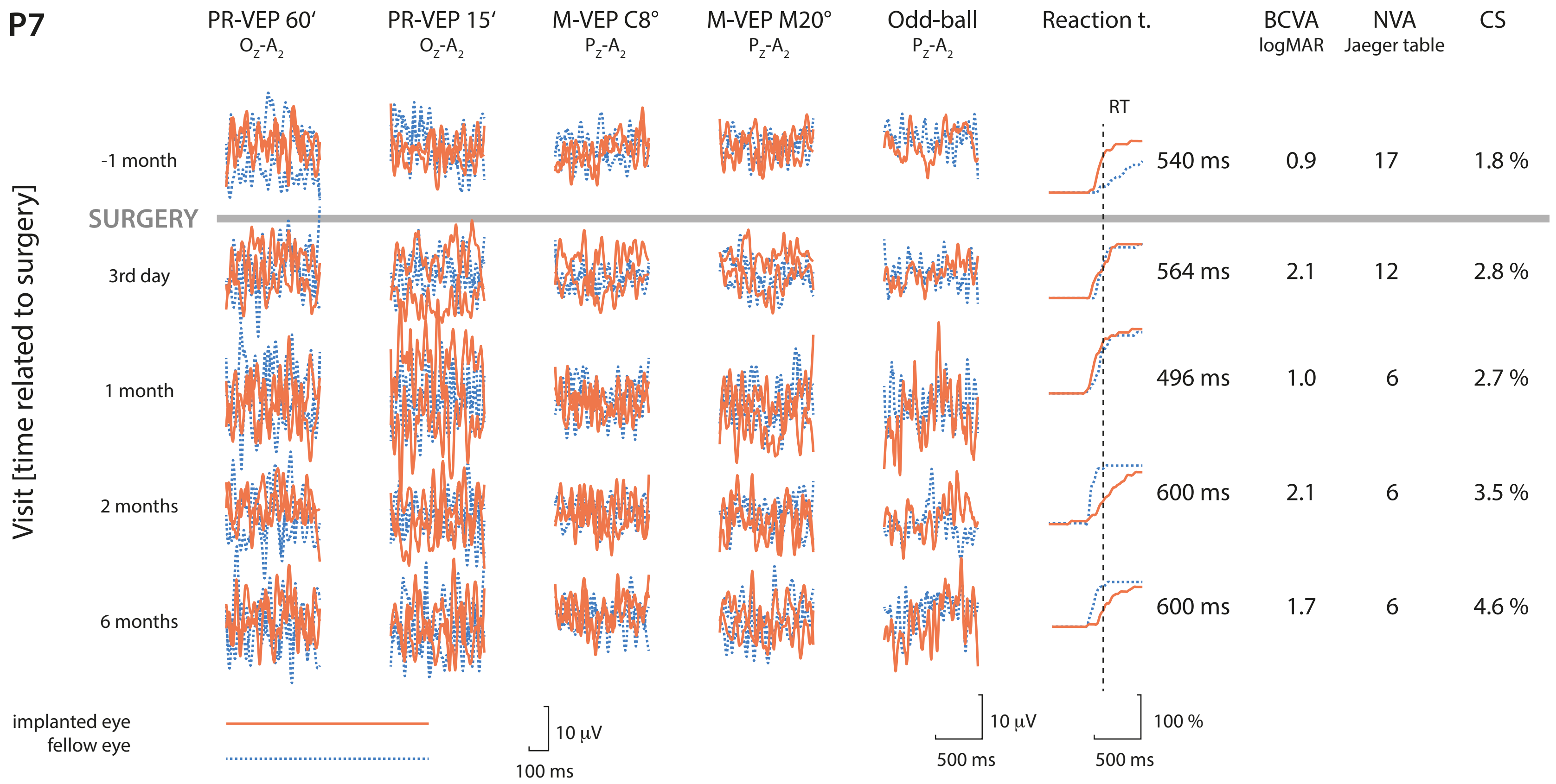
P7
PR-VEP 60′
O_Z-A_2
PR-VEP 15′
O_Z-A_2
M-VEP C8°
P_Z-A_2
M-VEP M20°
P_Z-A_2
Odd-ball
P_Z-A_2
Reaction t.
BCVA
logMAR
NVA
Jaeger table
CS
Visit [time related to surgery]
RT
-1 month
540 ms
0.9
17
1.8 %
SURGERY
3rd day
564 ms
2.1
12
2.8 %
1 month
496 ms
1.0
6
2.7 %
2 months
600 ms
2.1
6
3.5 %
6 months
600 ms
1.7
6
4.6 %
implanted eye
fellow eye
10 μV
100 ms
10 μV
500 ms
100 %
500 ms

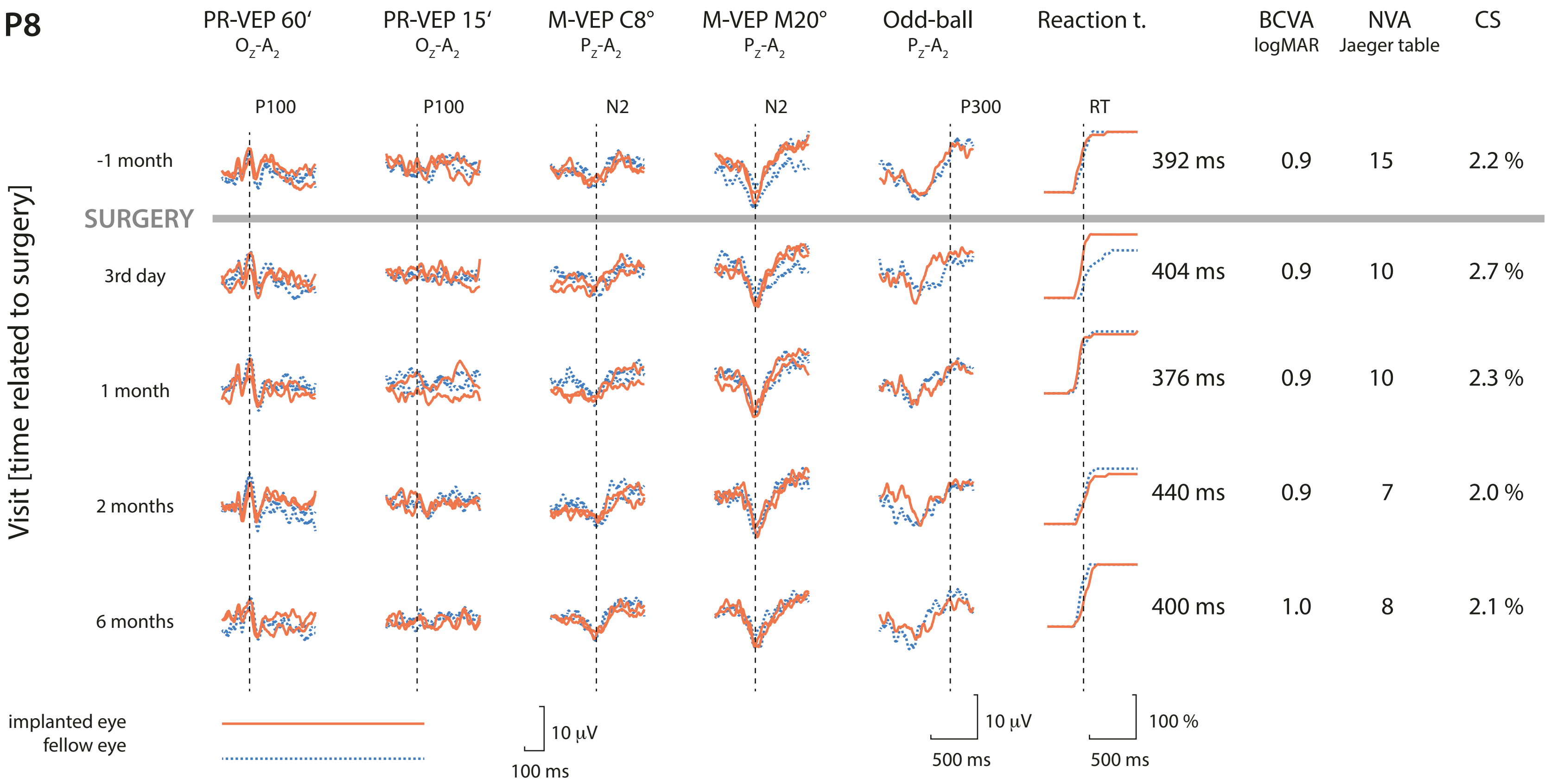

P8
PR-VEP 60′
O_Z-A_2
PR-VEP 15′
O_Z-A_2
M-VEP C8°
P_Z-A_2
M-VEP M20°
P_Z-A_2
Odd-ball
P_Z-A_2
Reaction t.
BCVA
logMAR
NVA
Jaeger table
CS
P100
P100
N2
N2
P300
RT
Visit [time related to surgery]
-1 month
392 ms
0.9
15
2.2 %
SURGERY
3rd day
404 ms
0.9
10
2.7 %
1 month
376 ms
0.9
10
2.3 %
2 months
440 ms
0.9
7
2.0 %
6 months
400 ms
1.0
8
2.1 %
implanted eye
fellow eye
10 μV
100 ms
10 μV
500 ms
100 %
500 ms

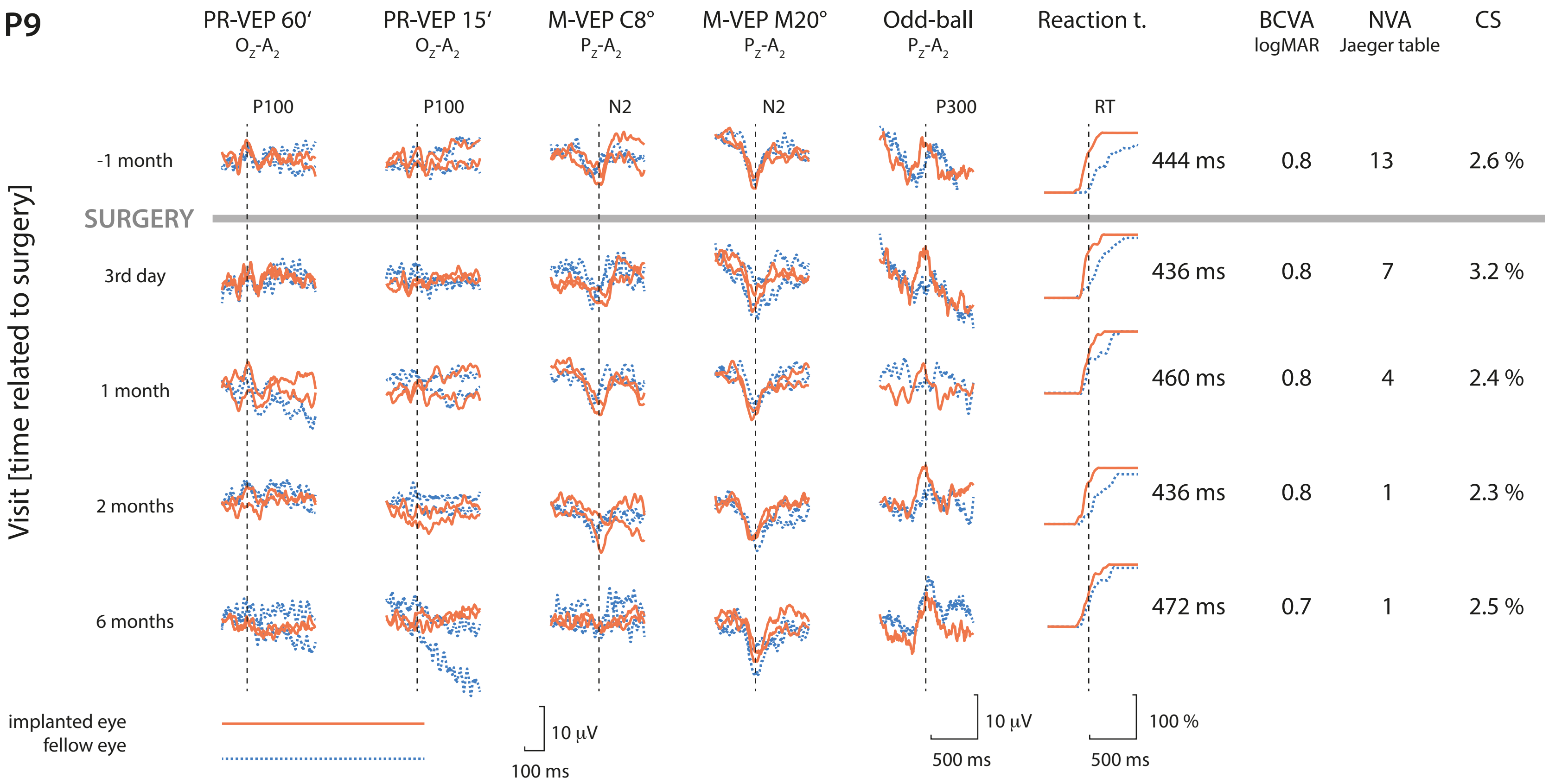

P9
PR-VEP 60′
O_Z-A_2
PR-VEP 15′
O_Z-A_2
M-VEP C8°
P_Z-A_2
M-VEP M20°
P_Z-A_2
Odd-ball
P_Z-A_2
Reaction t.
BCVA
logMAR
NVA
Jaeger table
CS
P100
P100
N2
N2
P300
RT
Visit [time related to surgery]
-1 month
444 ms
0.8
13
2.6 %
SURGERY
3rd day
436 ms
0.8
7
3.2 %
1 month
460 ms
0.8
4
2.4 %
2 months
436 ms
0.8
1
2.3 %
6 months
472 ms
0.7
1
2.5 %
implanted eye
fellow eye
10 μV
100 ms
10 μV
500 ms
100 %
500 ms

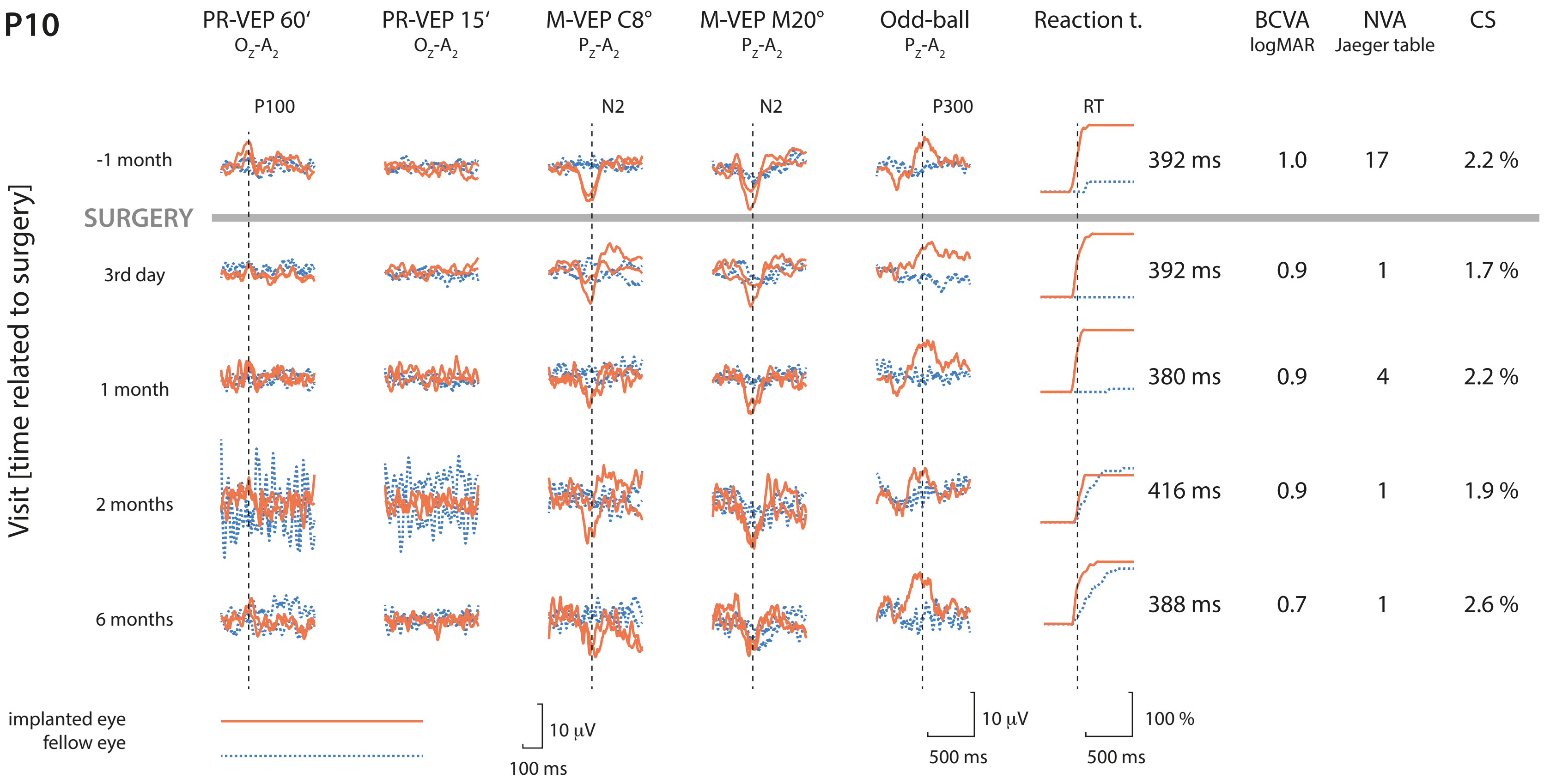

P10
PR-VEP 60′
O_Z-A_2
PR-VEP 15′
O_Z-A_2
M-VEP C8°
P_Z-A_2
M-VEP M20°
P_Z-A_2
Odd-ball
P_Z-A_2
Reaction t.
BCVA
logMAR
NVA
Jaeger table
CS
P100
N2
N2
P300
RT
Visit [time related to surgery]
-1 month
392 ms
1.0
17
2.2 %
SURGERY
3rd day
392 ms
0.9
1
1.7 %
1 month
380 ms
0.9
4
2.2 %
2 months
416 ms
0.9
1
1.9 %
6 months
388 ms
0.7
1
2.6 %
implanted eye
fellow eye
10 μV
100 ms
10 μV
500 ms
100 %
500 ms

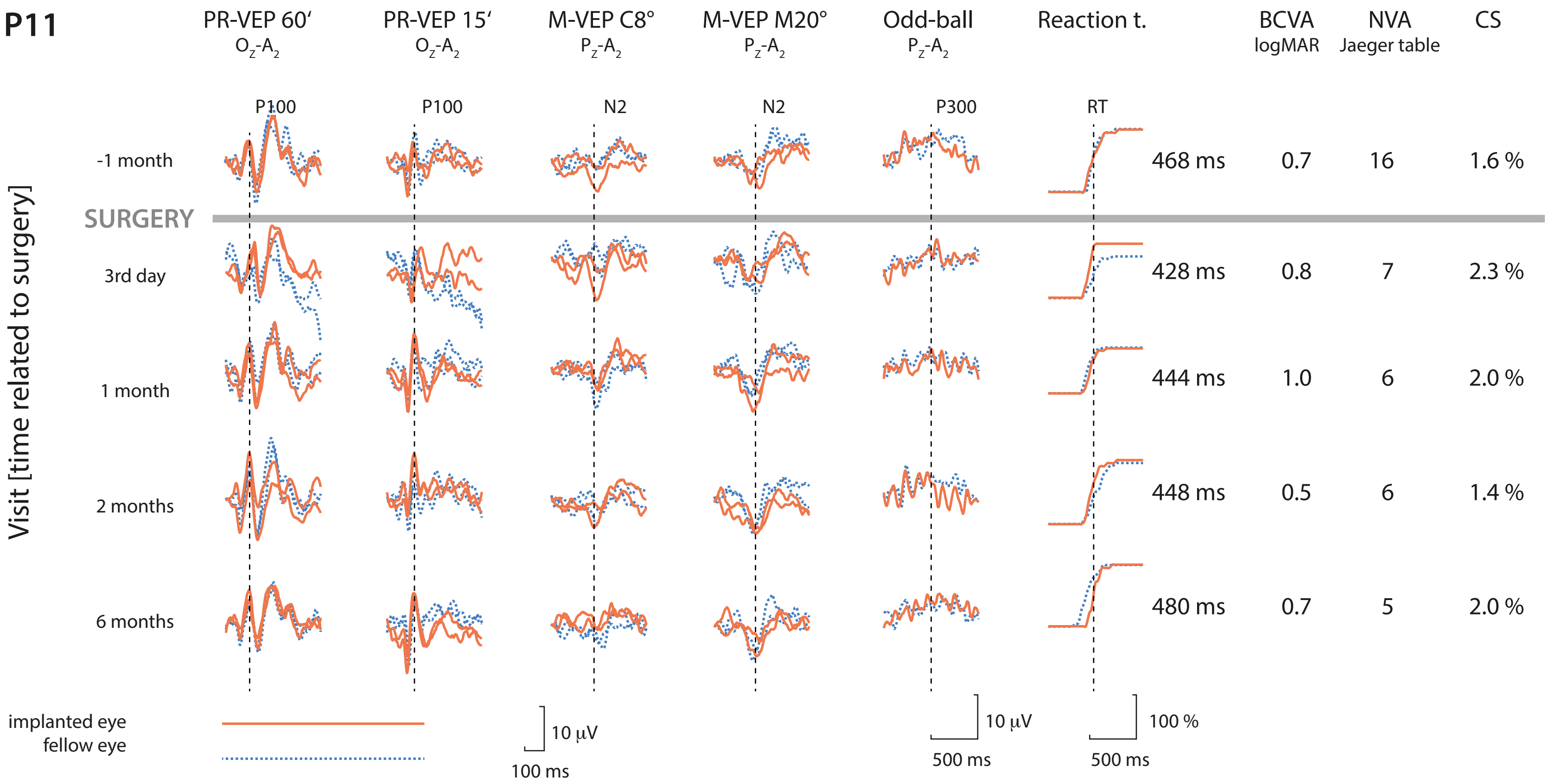
P11
PR-VEP 60‘
O_Z-A_2
PR-VEP 15‘
O_Z-A_2
M-VEP C8°
P_Z-A_2
M-VEP M20°
P_Z-A_2
Odd-ball
P_Z-A_2
Reaction t.
BCVA
logMAR
NVA
Jaeger table
CS
P100
P100
N2
N2
P300
RT
Visit [time related to surgery]
-1 month
468 ms
0.7
16
1.6 %
SURGERY
3rd day
428 ms
0.8
7
2.3 %
1 month
444 ms
1.0
6
2.0 %
2 months
448 ms
0.5
6
1.4 %
6 months
480 ms
0.7
5
2.0 %
implanted eye
fellow eye
10 µV
100 ms
10 µV
500 ms
100 %
500 ms

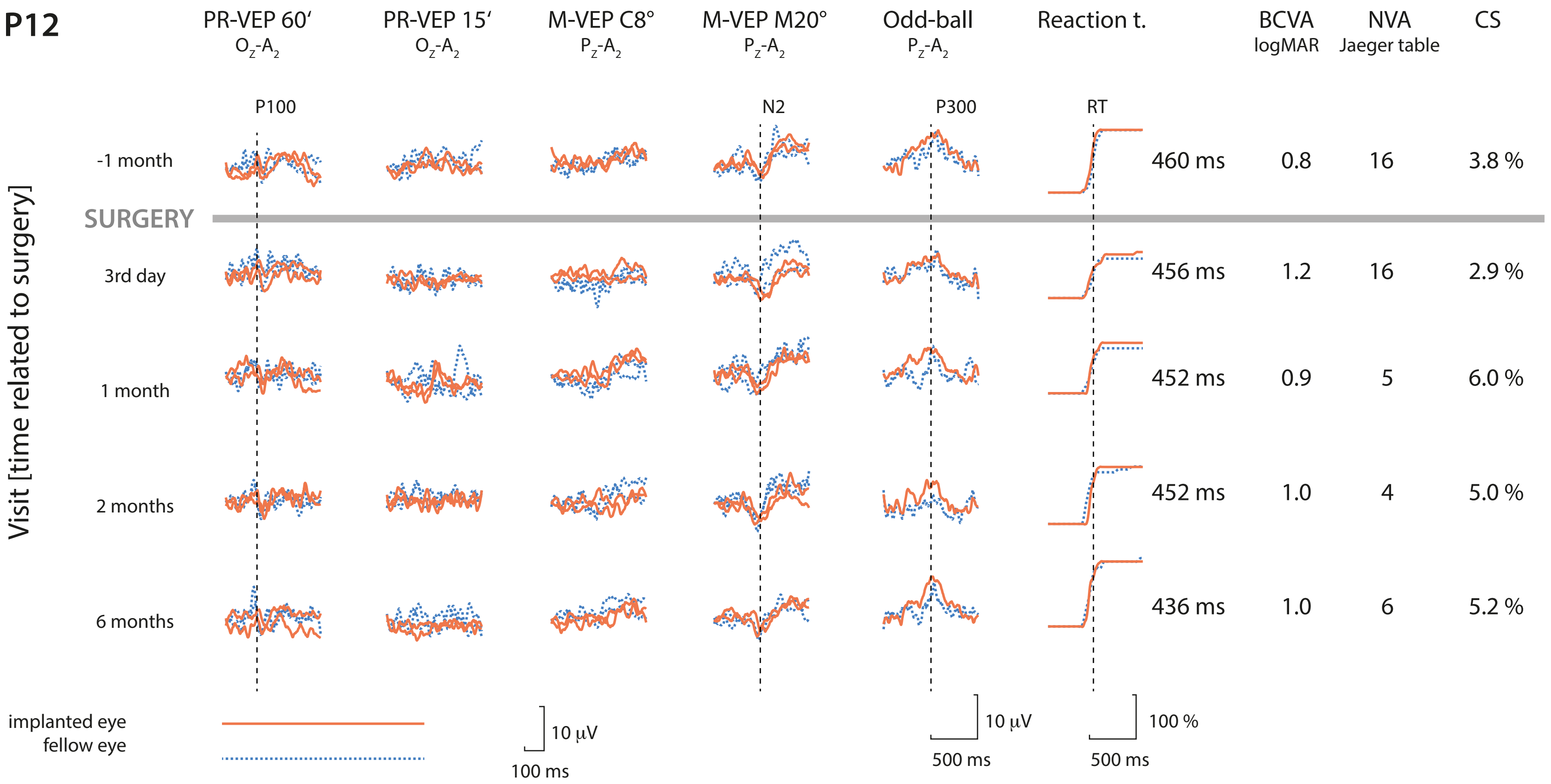

P12
PR-VEP 60′
O_Z-A_2
PR-VEP 15′
O_Z-A_2
M-VEP C8°
P_Z-A_2
M-VEP M20°
P_Z-A_2
Odd-ball
P_Z-A_2
Reaction t.
BCVA
logMAR
NVA
Jaeger table
CS
P100
N2
P300
RT
Visit [time related to surgery]
-1 month
460 ms
0.8
16
3.8 %
SURGERY
3rd day
456 ms
1.2
16
2.9 %
1 month
452 ms
0.9
5
6.0 %
2 months
452 ms
1.0
4
5.0 %
6 months
436 ms
1.0
6
5.2 %
implanted eye
fellow eye
10 μV
100 ms
10 μV
500 ms
100 %
500 ms

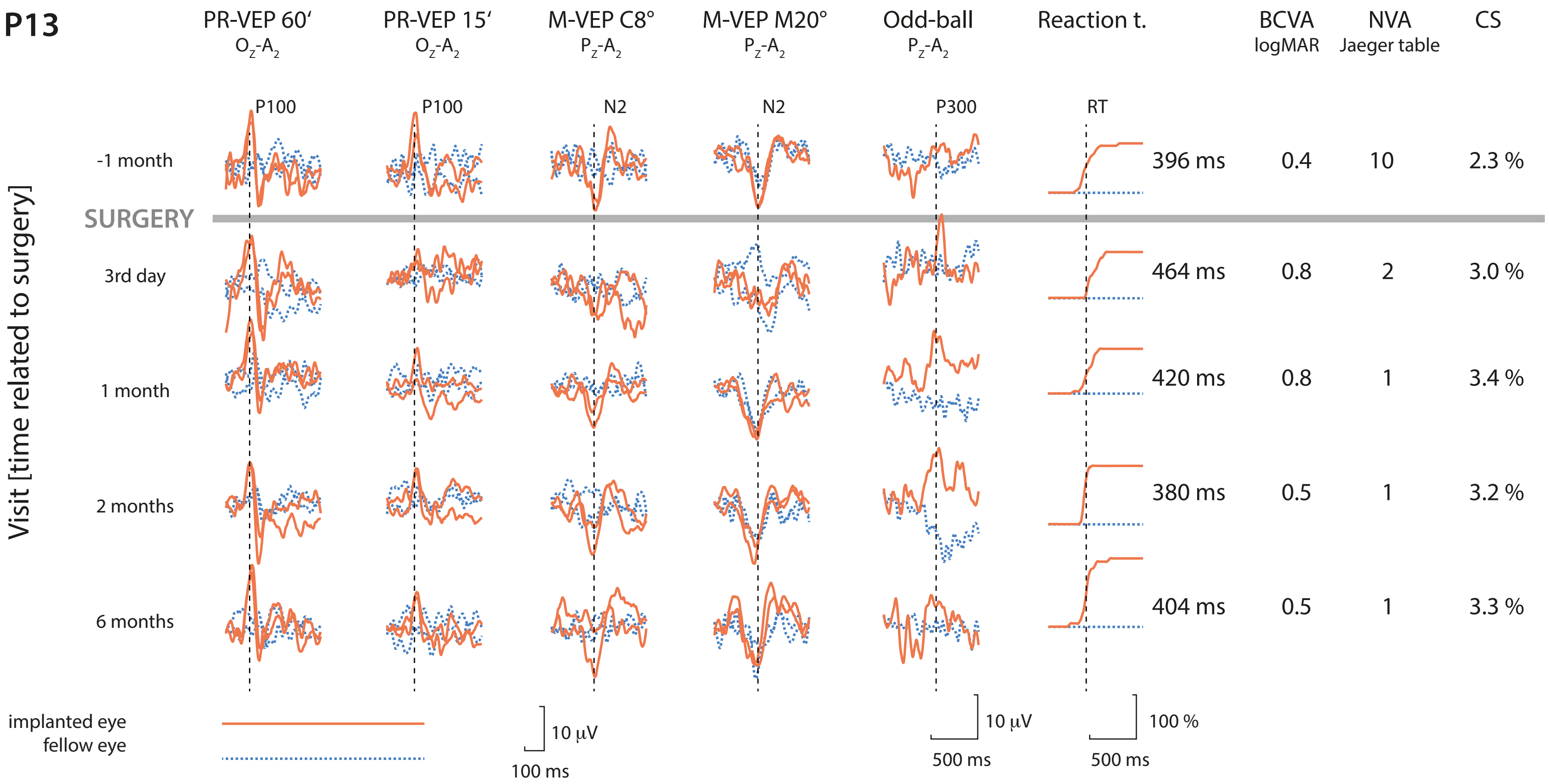

P13
PR-VEP 60′
O_Z-A_2
PR-VEP 15′
O_Z-A_2
M-VEP C8°
P_Z-A_2
M-VEP M20°
P_Z-A_2
Odd-ball
P_Z-A_2
Reaction t.
BCVA
logMAR
NVA
Jaeger table
CS
P100
P100
N2
N2
P300
RT
Visit [time related to surgery]
-1 month
396 ms
0.4
10
2.3 %
SURGERY
3rd day
464 ms
0.8
2
3.0 %
1 month
420 ms
0.8
1
3.4 %
2 months
380 ms
0.5
1
3.2 %
6 months
404 ms
0.5
1
3.3 %
implanted eye
fellow eye
10 µV
100 ms
10 µV
500 ms
100 %
500 ms

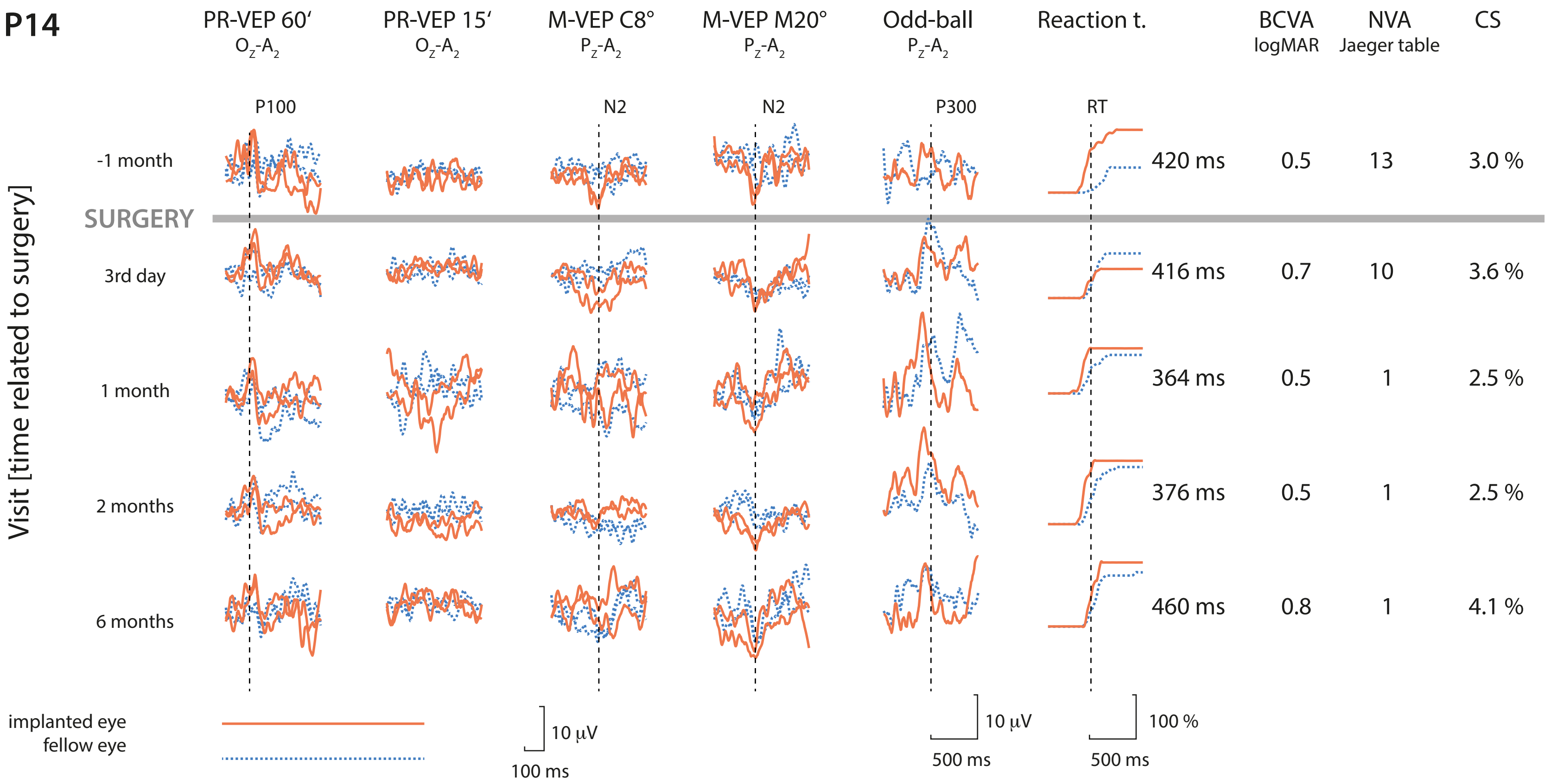
P14
PR-VEP 60‘
Oz-A2
PR-VEP 15‘
Oz-A2
M-VEP C8°
Pz-A2
M-VEP M20°
Pz-A2
Odd-ball
Pz-A2
Reaction t.
BCVA
logMAR
NVA
Jaeger table
CS
P100
N2
N2
P300
RT
Visit [time related to surgery]
-1 month
420 ms
0.5
13
3.0 %
SURGERY
3rd day
416 ms
0.7
10
3.6 %
1 month
364 ms
0.5
1
2.5 %
2 months
376 ms
0.5
1
2.5 %
6 months
460 ms
0.8
1
4.1 %
implanted eye
fellow eye
10 µV
100 ms
10 µV
500 ms
100 %
500 ms

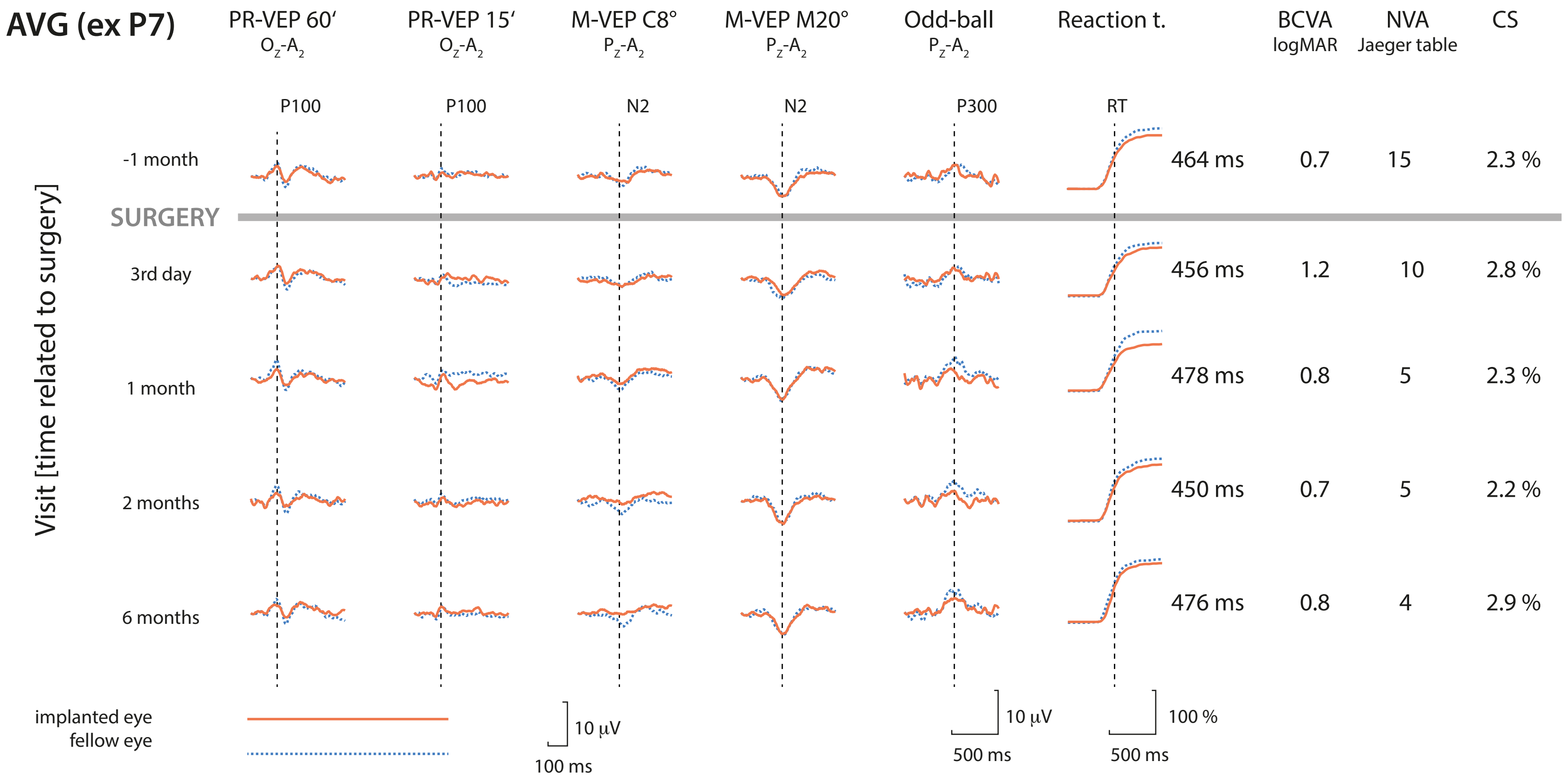

AVG (ex P7)
PR-VEP 60′
O_Z-A_2
PR-VEP 15′
O_Z-A_2
M-VEP C8°
P_Z-A_2
M-VEP M20°
P_Z-A_2
Odd-ball
P_Z-A_2
Reaction t.
BCVA
logMAR
NVA
Jaeger table
CS
P100
P100
N2
N2
P300
RT
Visit [time related to surgery]
-1 month
464 ms
0.7
15
2.3 %
SURGERY
3rd day
456 ms
1.2
10
2.8 %
1 month
478 ms
0.8
5
2.3 %
2 months
450 ms
0.7
5
2.2 %
6 months
476 ms
0.8
4
2.9 %
implanted eye
fellow eye
10 µV
100 ms
10 µV
500 ms
100 %
500 ms
