# Supplementary material for: Vision before and after scharioth macular lens implantation in patients with AMD: an electrophysiological study
Source: Doc Ophthalmol. 2021 Jan 3;143(1):17–31. doi: 10.1007/s10633-020-09814-8 (PMC8266777; doi:10.1007/s10633-020-09814-8)
Supplement: Supplementary file 2 — (PDF 493 kb) [file 10633_2020_9814_MOESM2_ESM.pdf]

Vision before and after Scharioth macular lens implantation in patients with AMD: an electrophysiological study

Kremláček Jan, Nekolová Jana, Středová Markéta, Langrová Jana, Szanyi Jana, Kuba Miroslav, Kubová Zuzana, Vít František,
Voda Petr, Veselá Martina, Jirásková Naďa

Supplementary material part B

Tables and graphs for all parameters

Every plot combines boxplots summarizing a particular parameter grouped by visit. The individual measurements are depicted as semitransparent gray points connected by dotted lines to depict within-patient relations.

For the trend analysis, a within-subject slope between visits was tested against zero (null h. = there is no slope). The slope was calculated without the second visit (the one immediately following the implantation).

Table of Contents

Supplementary material 1
- PR-VEP 60’ P100 peak time 2
- PR-VEP 60’ P100 amplitude 3
- PR-VEP 15’ P100 peak time 4
- PR-VEP 15’ P100 amplitude 5
- M-VEP C8° N2 peak time 6
- M-VEP C8° N2 amplitude 7
- M-VEP M20° N2 peak time 8
- M-VEP M20° N2 amplitude 9
- Odd-ball P300 peak time 10
- Odd-ball P300 amplitude 11
- Reaction time 12
- BCVA 13
- NVA 14
- NVA +6D 15
- CS Land 16

PR-VEP 60’ P100 peak time

Descriptive statistcs

	-1 month	3rd day	1 month	2 months	6 months
n	13	11	11	12	11
PR-VEP 60’ P100 peak time [ms]	128 (118; 132)	133 (126; 136.5)	130 (122; 135.5)	129.5 (113.5; 133)	129 (119; 136)

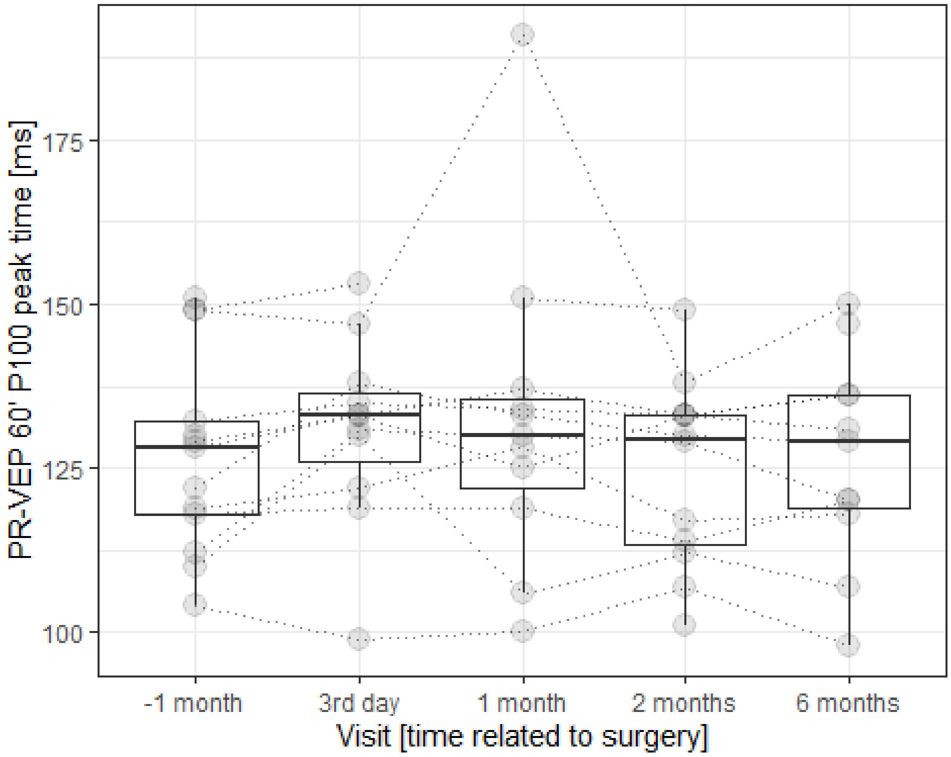

Trend analysis

	n / med (25q; 75q)
n	10
PR-VEP 60’ P100 peak time [ms] /year	-0.3 (-5.3; 5.9)

Anderson-Darling test: normality = TRUE

Two tail tests

NOT different from zero, ttest p= 0.7804685

Equivalent effect size and confidence limits, d = 0.13 [-0.75 1]

Power of study with aforementioned effect, pwr = 0.06

PR-VEP 60’ P100 amplitude

Descriptive statistcs

	-1 month	3rd day	1 month	2 months	6 months
n	14	14	14	14	14
PR-VEP 60’ P100 amplitude [uV]	5 (3.6; 7)	4.4 (2.8; 7.8)	4.4 (3.4; 6.9)	6.1 (2.9; 6.5)	4.6 (4.1; 8)

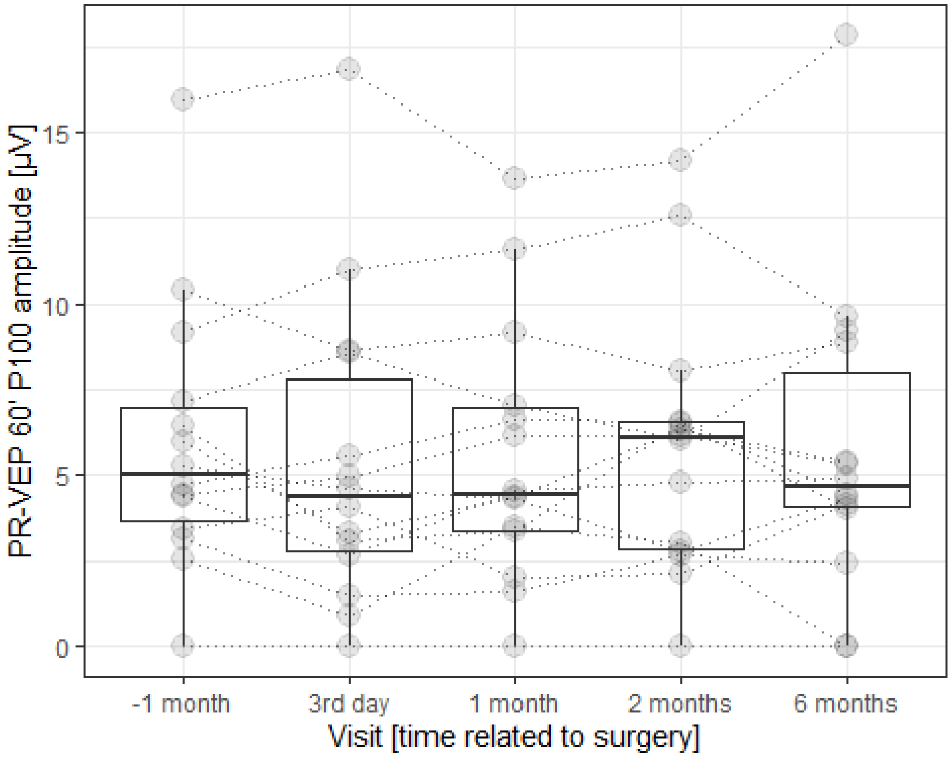

Trend analysis

	n / med (25q; 75q)
n	14
PR-VEP 60’ P100 amplitude [uV] /year	-0.1 (-1.5; 0.9)

Anderson-Darling test: normality = TRUE

Two tail tests

NOT different from zero, ttest p= 0.9862365

Equivalent effect size and confidence limits, d = 0.01 [-0.73 0.75]

Power of study with aforementioned effect, pwr = 0.05

PR-VEP 15’ P100 peak time

Descriptive statistcs

	-1 month	3rd day	1 month	2 months	6 months
n	7	8	10	5	9
PR-VEP 15’ P100 peak time [ms]	147 (135; 151)	152 (147.8; 157.8)	143.5 (141.2; 150.5)	140 (137; 145)	161 (141; 166)

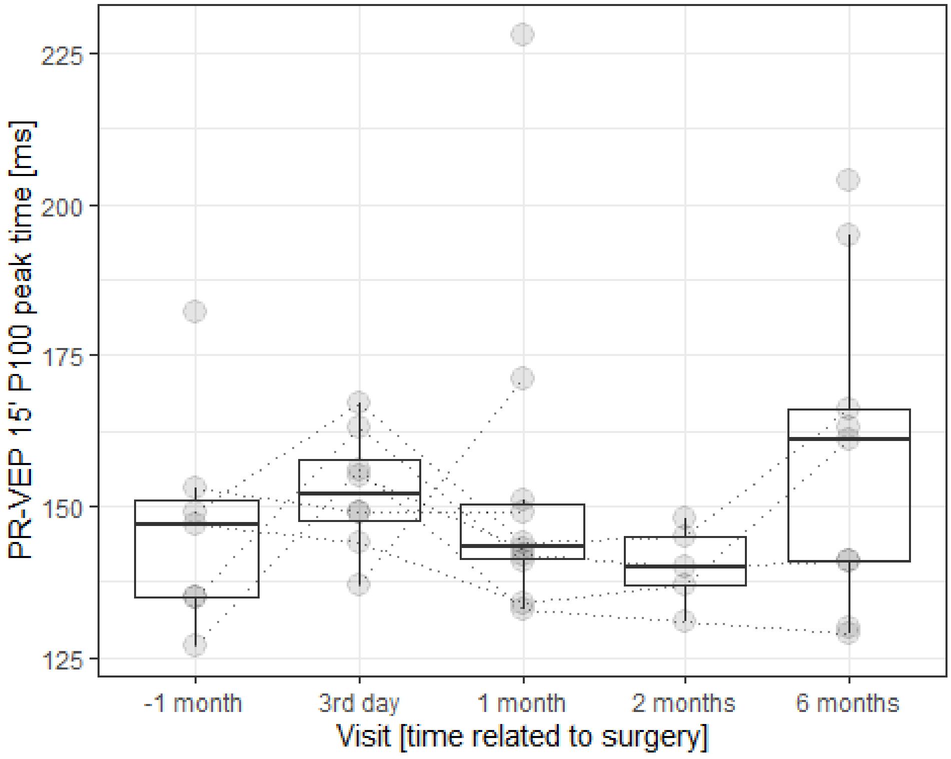

Trend analysis

	n / med (25q; 75q)
n	4
PR-VEP 15’ P100 peak time [ms] /year	21.2 (6; 36)

Anderson-Darling test: normality = FALSE

Two tail tests

NOT different from , Wilcoxon p= 0.125

Equivalent effect size and confidence limits, d = 1.26 [-0.26 2.78]

Power of study with aforementioned effect, pwr = 0.32

PR-VEP 15’ P100 amplitude

Descriptive statistcs

	-1 month	3rd day	1 month	2 months	6 months
n	14	14	14	14	14
PR-VEP 15’ P100 amplitude [uV]	2 (0.9; 3.5)	1.9 (1.1; 4.6)	2.9 (0.6; 6.4)	0.9 (0; 3.9)	3.3 (0.5; 5.2)

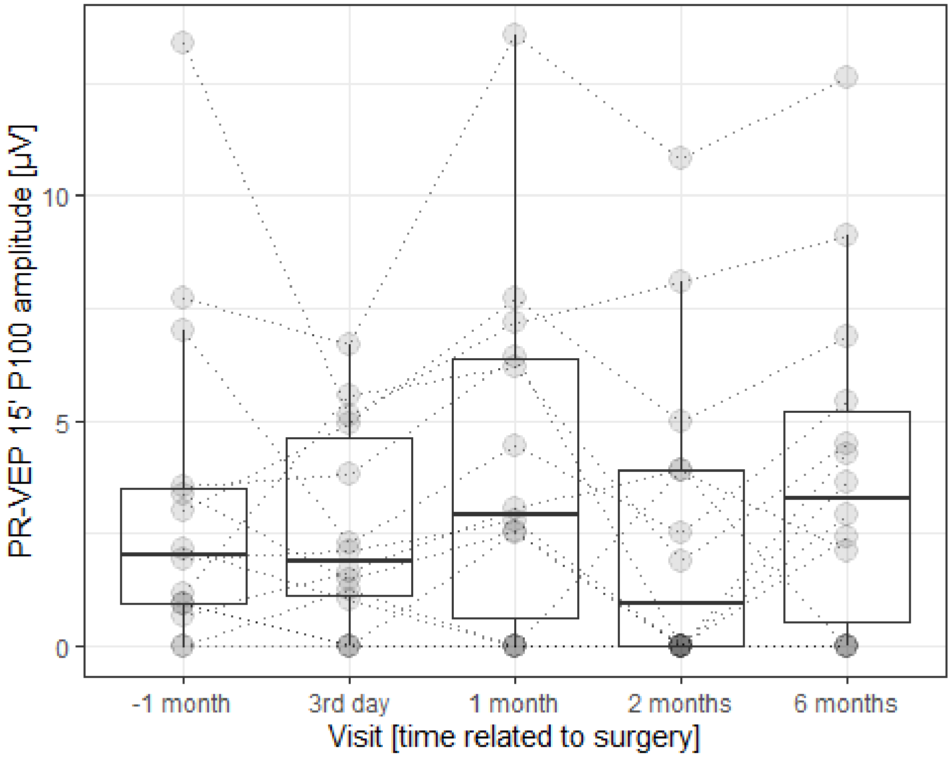

Trend analysis

	n / med (25q; 75q)
n	14
PR-VEP 15’ P100 amplitude [uV] /year	-0.1 (-1.4; 3.2)

Anderson-Darling test: normality = TRUE

Two tail tests

NOT different from zero, ttest p= 0.4318001

Equivalent effect size and confidence limits, d = 0.3 [-0.44 1.05]

Power of study with aforementioned effect, pwr = 0.12

M-VEP C8° N2 peak time

Descriptive statistcs

	-1 month	3rd day	1 month	2 months	6 months
n	8	8	6	9	8
M-VEP C8° N2 peak time [ms]	210 (185.8; 220)	194 (185; 205)	205 (193; 226)	199 (193; 228)	211.5 (202; 219)

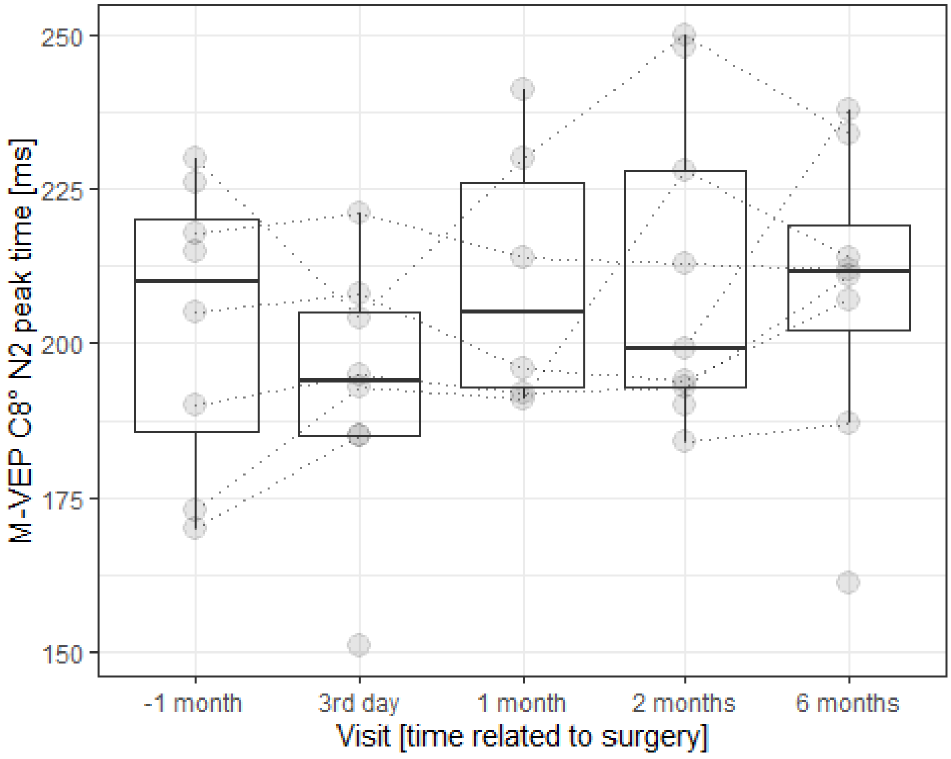

Trend analysis

	n / med (25q; 75q)
n	5
M-VEP C8° N2 peak time [ms] /year	7.3 (6.5; 43)

Anderson-Darling test: normality = FALSE

Two tail tests

NOT different from , Wilcoxon p= 0.3125

Equivalent effect size and confidence limits, d = 0.68 [-0.59 1.96]

Power of study with aforementioned effect, pwr = 0.16

M-VEP C8° N2 amplitude

Descriptive statistcs

	-1 month	3rd day	1 month	2 months	6 months
n	14	14	14	14	14
M-VEP C8° N2 amplitude [uV]	4.1 (0; 6.9)	2.9 (0; 6.3)	2.7 (0; 6.4)	3.2 (0; 6)	3.2 (0; 5.4)

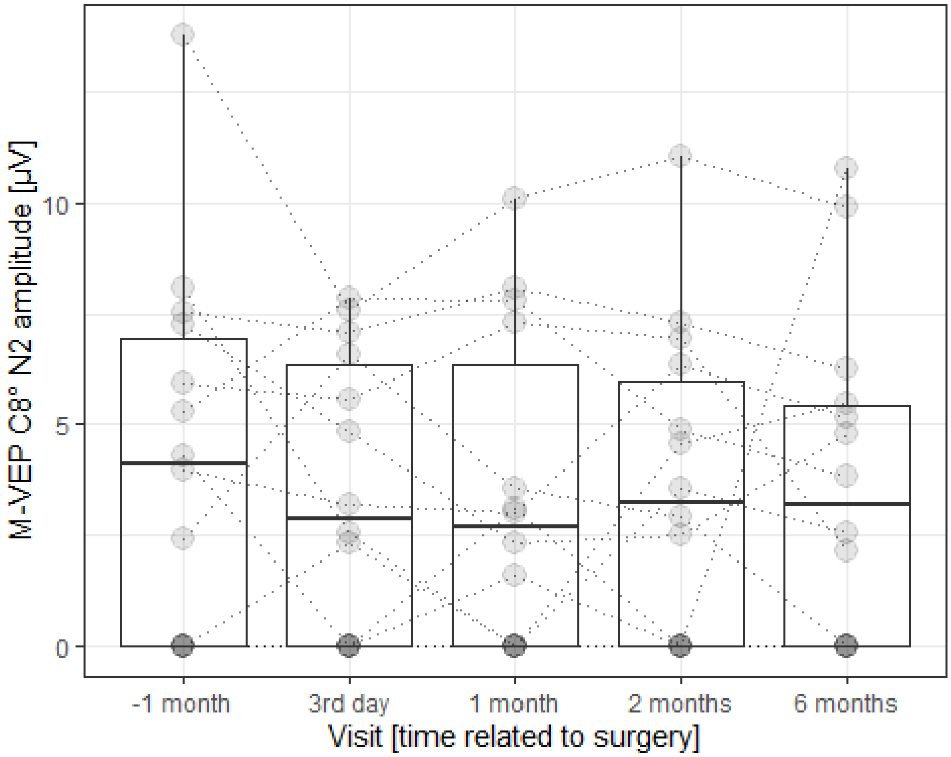

Trend analysis

	n / med (25q; 75q)
n	14
M-VEP C8° N2 amplitude [uV] /year	-1.5 (-4.6; 0)

Anderson-Darling test: normality = FALSE

Two tail tests

NOT different from , Wilcoxon p= 0.2240154

Equivalent effect size and confidence limits, d = 0.47 [-0.28 1.22]

Power of study with aforementioned effect, pwr = 0.22

M-VEP M20° N2 peak time

Descriptive statistcs

	-1 month	3rd day	1 month	2 months	6 months
n	14	11	13	12	13
M-VEP M20° N2 peak time [ms]	191.5 (186.5; 214.5)	203 (192.5; 225.5)	191 (185; 211)	198.5 (188; 205.5)	205 (187; 218)

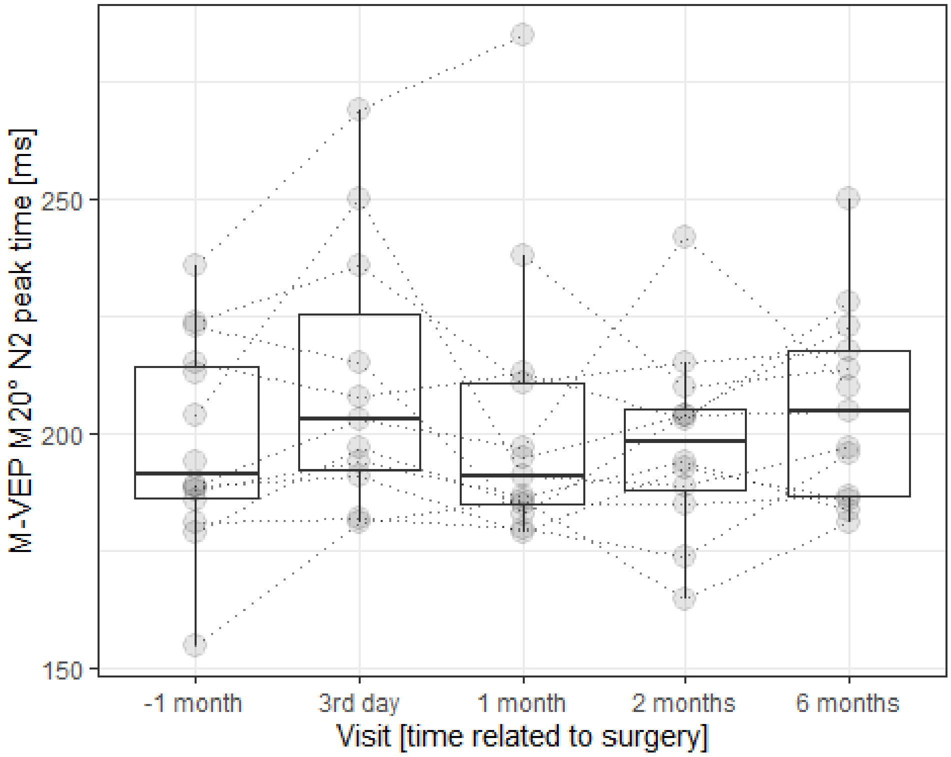

Trend analysis

	n / med (25q; 75q)
n	12
M-VEP M20° N2 peak time [ms] /year	14.1 (-4.7; 26.8)

Anderson-Darling test: normality = TRUE

Two tail tests

SIGNIFICANT difference from zero, ttest p= 0.03554169

Equivalent effect size and confidence limits, d = 0.91 [0.07 1.76]

Power of study with aforementioned effect, pwr = 0.57

M-VEP M20° N2 amplitude

Descriptive statistcs

	-1 month	3rd day	1 month	2 months	6 months
n	14	14	14	14	14
M-VEP M20° N2 amplitude [uV]	6.9 (5.7; 7.9)	5.6 (3.9; 7)	6.8 (5.8; 8.5)	5.4 (4.6; 7.9)	6.6 (4.8; 7.4)

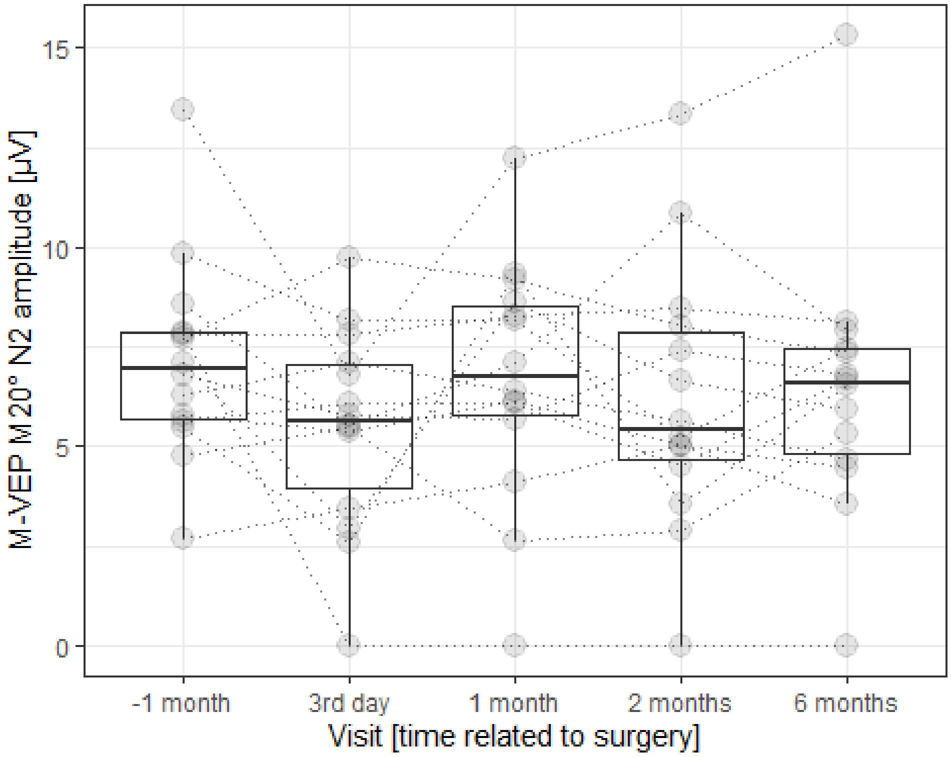

Trend analysis

	n / med (25q; 75q)
n	14
M-VEP M20° N2 amplitude [uV] /year	-0.1 (-2.8; 1.3)

Anderson-Darling test: normality = TRUE

Two tail tests

NOT different from zero, ttest p= 0.3247922

Equivalent effect size and confidence limits, d = 0.38 [-0.37 1.13]

Power of study with aforementioned effect, pwr = 0.16

Odd-ball P300 peak time

Descriptive statistcs

	-1 month	3rd day	1 month	2 months	6 months
n	14	11	13	11	11
Odd-ball P300 peak time [ms]	520 (506; 595)	508 (466; 562)	524 (492; 580)	492 (472; 560)	504 (470; 539.5)

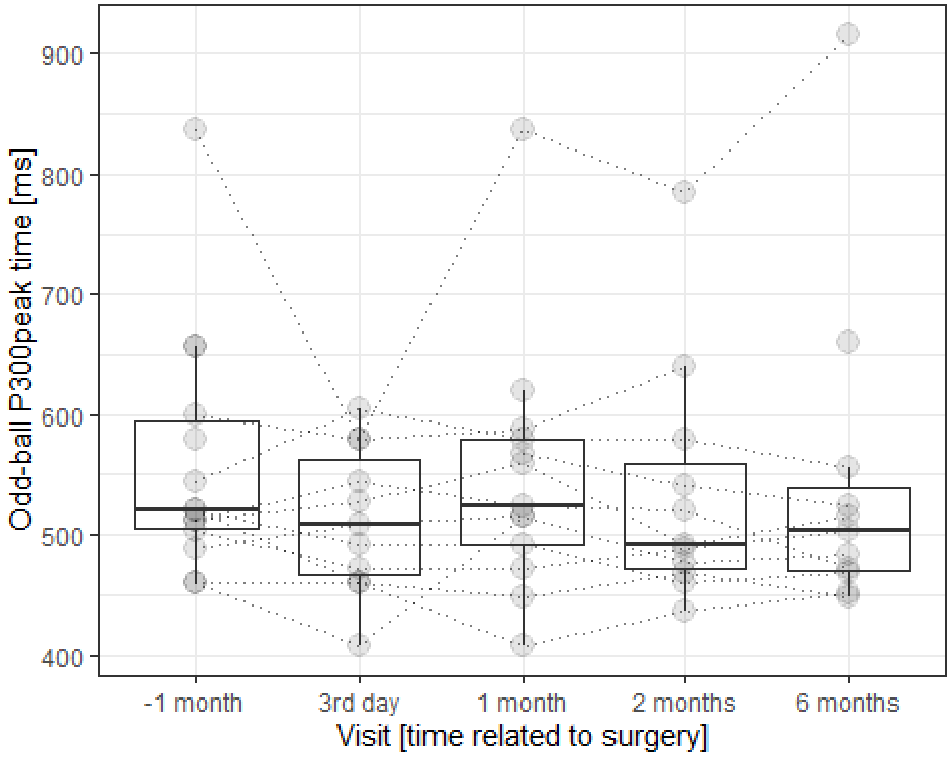

Trend analysis

	n / med (25q; 75q)
n	10
Odd-ball P300 peak time [ms] /year	-20.2 (-89.3; 7.7)

Anderson-Darling test: normality = TRUE

Two tail tests

NOT different from zero, ttest p= 0.3867805

Equivalent effect size and confidence limits, d = 0.4 [-0.49 1.28]

Power of study with aforementioned effect, pwr = 0.14

Odd-ball P300 amplitude

Descriptive statistcs

	-1 month	3rd day	1 month	2 months	6 months
n	14	14	14	14	13
Odd-ball P300 amplitude [uV]	11.1 (7.6; 13.4)	10.2 (5.4; 12.9)	9.7 (7; 15.9)	11.7 (4.4; 14.4)	10.6 (5.2; 20)

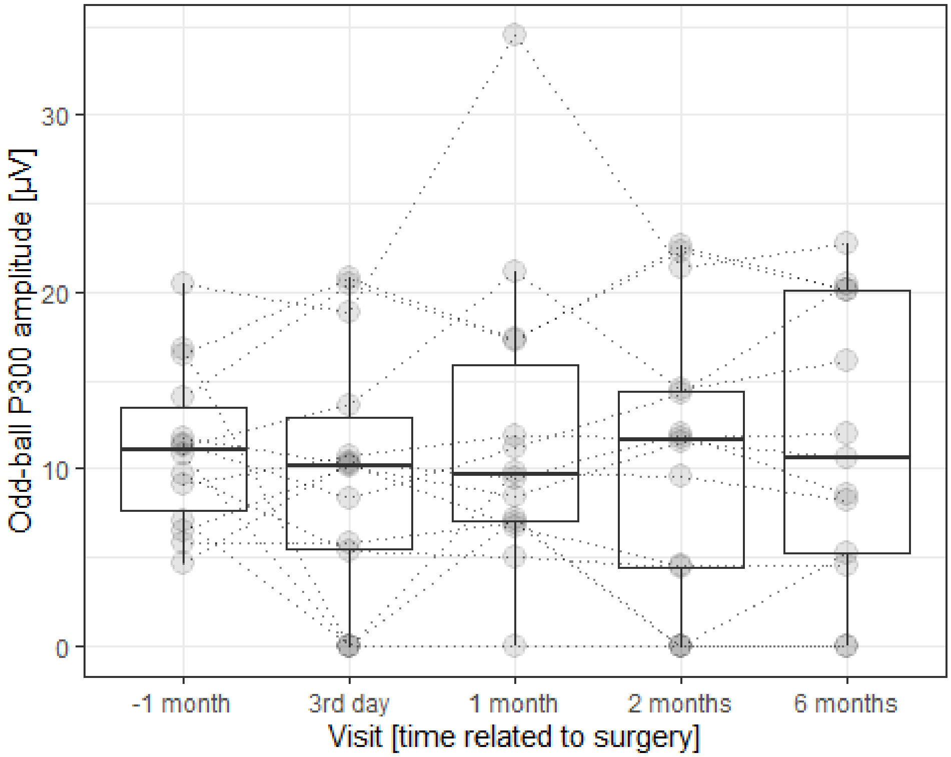

Trend analysis

	n / med (25q; 75q)
n	13
Odd-ball P300 amplitude [uV] /year	3.6 (-4.2; 6.7)

Anderson-Darling test: normality = TRUE

Two tail tests

NOT different from zero, ttest p= 0.9720423

Equivalent effect size and confidence limits, d = 0.01 [-0.75 0.78]

Power of study with aforementioned effect, pwr = 0.05

Reaction time

Descriptive statistcs

	-1 month	3rd day	1 month	2 months	6 months
n	14	13	14	14	14
Reaction time [ms]	464 (425; 496)	456 (428; 540)	478 (426; 524)	450 (433; 542)	476 (442; 543)

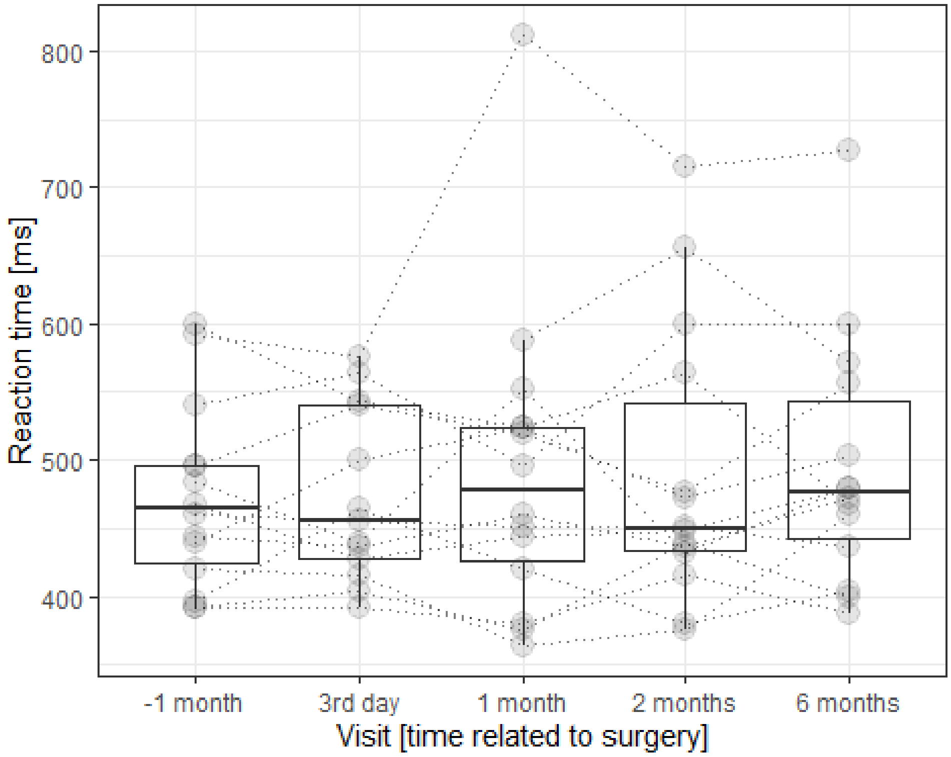

Trend analysis

	n / med (25q; 75q)
n	14
Reaction time [ms] /year	31.6 (-10.5; 77)

Anderson-Darling test: normality = TRUE

Two tail tests

NOT different from zero, ttest p= 0.1762475

Equivalent effect size and confidence limits, d = 0.53 [-0.23 1.28]

Power of study with aforementioned effect, pwr = 0.27

BCVA

Descriptive statistcs

	-1 month	3rd day	1 month	2 months	6 months
n	14	14	14	14	14
BCVA [logMAR]	0.7 (0.5; 0.8)	1.2 (0.8; 1.2)	0.8 (0.5; 1)	0.7 (0.5; 0.8)	0.8 (0.7; 0.8)

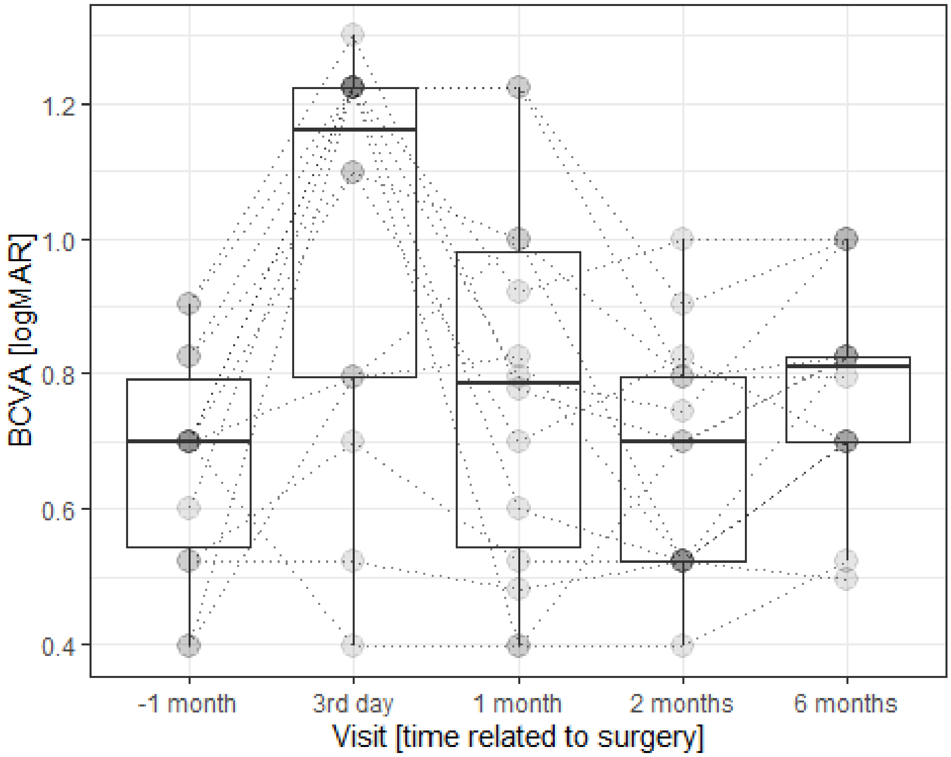

Trend analysis

	n / med (25q; 75q)
n	14
BCVA [logMAR] /year	0 (0; 0.4)

Anderson-Darling test: normality = FALSE

Two tail tests

NOT different from , Wilcoxon p= 0.2165527

Equivalent effect size and confidence limits, d = 0.48 [-0.27 1.23]

Power of study with aforementioned effect, pwr = 0.23

NVA

Descriptive statistcs

	-1 month	3rd day	1 month	2 months	6 months
n	14	14	14	14	14
NVA [JT]	15 (13.2; 16.8)	10 (6.2; 12)	4.5 (3; 6.8)	5 (1; 6)	3.5 (1; 6)

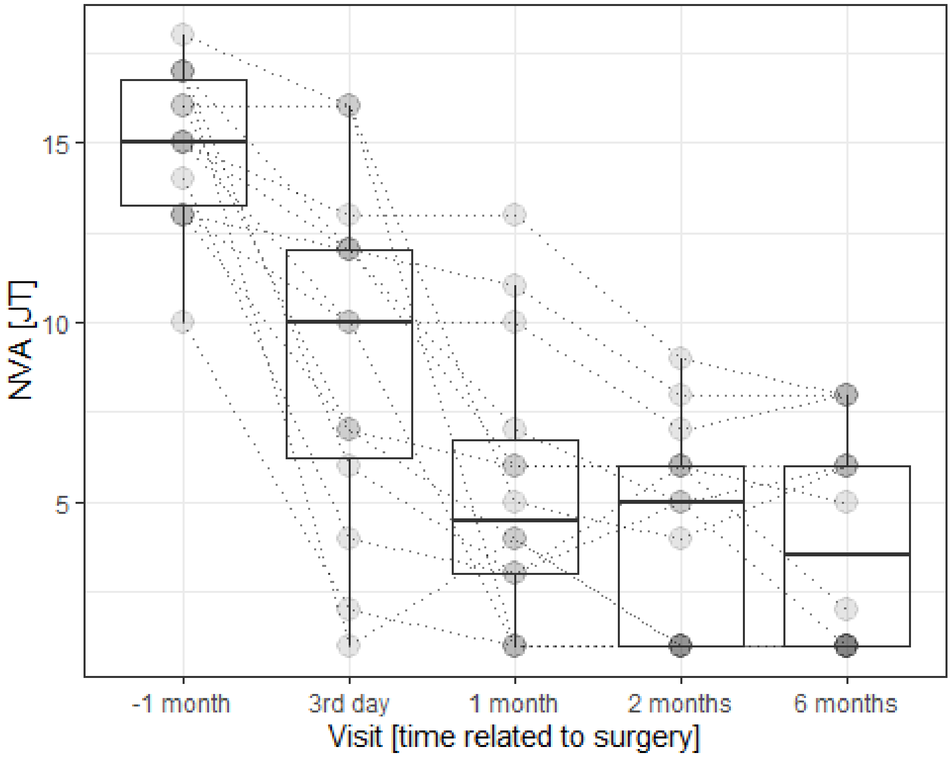

Trend analysis

	n / med (25q; 75q)
n	14
NVA [JT] /year	-12.7 (-17.1; -11.4)

Anderson-Darling test: normality = TRUE

Two tail tests

SIGNIFICANT difference from zero, ttest p= 3.711861e-08

Equivalent effect size and confidence limits, d = 2.91 [1.84 3.97]

Power of study with aforementioned effect, pwr = 1

NVA +6D

Descriptive statistcs

	-1 month	3rd day	1 month	2 months	6 months
n	14	14	14	14	14
NVA +6D [JT]	6 (3.2; 6.8)	10 (6.2; 12)	4.5 (3; 6.8)	5 (1; 6)	3.5 (1; 6)

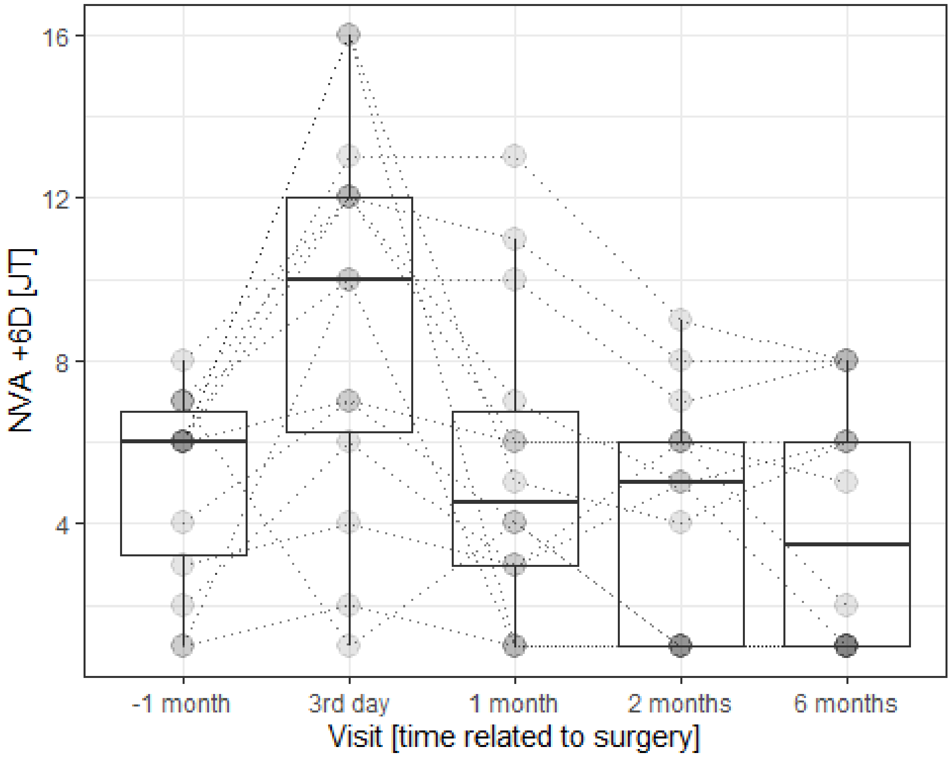

Trend analysis

	n / med (25q; 75q)
n	14
NVA +6D [JT] /year	-1 (-3; 0)

Anderson-Darling test: normality = FALSE

Two tail tests

SIGNIFICANT difference from zero, Wilcoxon p= 0.03667129

Equivalent effect size and confidence limits, d = 0.83 [0.06 1.6]

Power of study with aforementioned effect, pwr = 0.56

CS Land

Descriptive statistcs

	-1 month	3rd day	1 month	2 months	6 months
n	14	14	14	14	14
CS Land [%]	2.3 (1.7; 2.9)	2.8 (2.1; 3.2)	2.3 (2.1; 3.2)	2.2 (1.8; 3.4)	2.9 (2.1; 4.8)

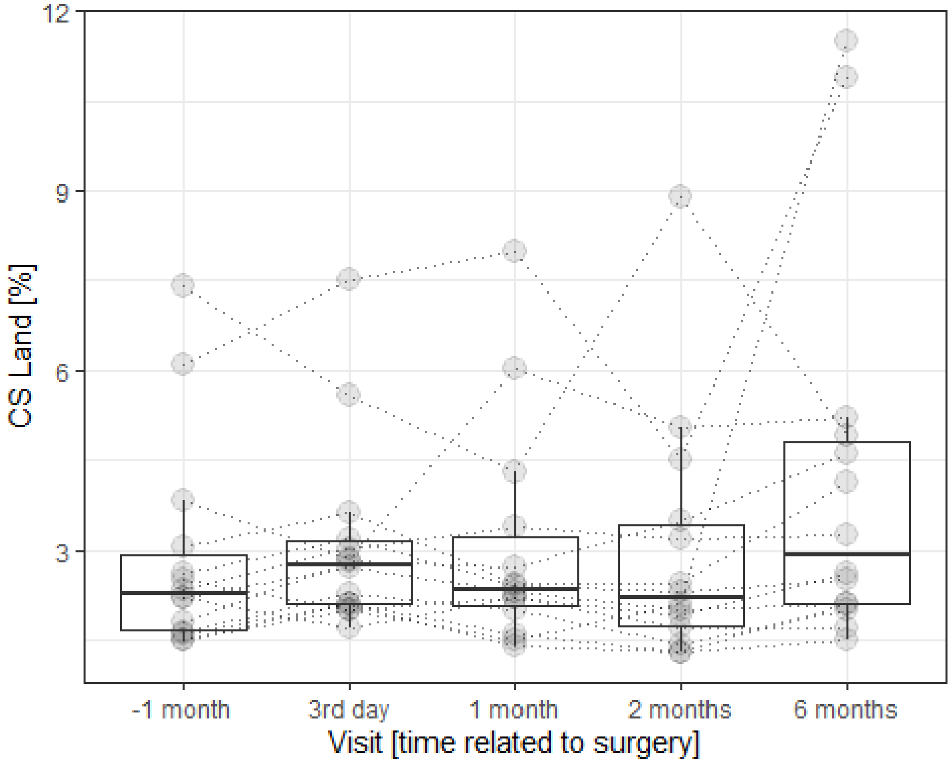

Trend analysis

	n / med (25q; 75q)
n	14
CS Land [%] /year	1 (-0.1; 1.7)

Anderson-Darling test: normality = FALSE

Two tail tests

NOT different from , Wilcoxon p= 0.09057617

Equivalent effect size and confidence limits, d = 0.66 [-0.1 1.43]

Power of study with aforementioned effect, pwr = 0.39
